# PT_f_‐SRiApt Targeting SCAF4‐POLR2A Interaction Suppresses Tumor Growth and Promotes Antitumor Immunity in Triple‐Negative Breast Cancer

**DOI:** 10.1002/advs.202500433

**Published:** 2025-06-27

**Authors:** Liyan Fei, Yichun Pan, Jie Zhai, Tongqing Li, Yuxin Zhou, Sheyu Zhang, Juan Wei, Qian Hu, Xueying Liu, Lu Guo, Weizhu Wu, Yong Wei, Qin Wu, Weihong Tan

**Affiliations:** ^1^ School of Chemistry and Materials Science University of Science and Technology of China Hefei 230026 P. R. China; ^2^ Department of Breast Surgery Zhejiang Cancer Hospital Hangzhou 310022 P. R. China; ^3^ The Key Laboratory of Zhejiang Province for Aptamers and Theranostics Hangzhou Institute of Medicine (HIM) Chinese Academy of Sciences Hangzhou 310022 P. R. China; ^4^ Department of Breast Surgery Ningbo Medical Center Lihuili Hospital Ningbo 315040 P. R. China; ^5^ School of Pharmaceutical Science and Technology Hangzhou Institute for Advanced Study University of Chinese Academy of Sciences Hangzhou 311300 P. R. China

**Keywords:** aptamers, cancers, personalized medicine, protein‐protein interactions, transcription factors

## Abstract

The interaction between SCAF4 and RNA polymerase II (POLR2A) is crucial for proper mRNA termination, with its dysregulation leading to truncated mRNAs and nonfunctional proteins, impairing cellular growth. Despite its potential relevance, the role of this interaction in triple‐negative breast cancer (TNBC) remains unexplored due to the lack of effective molecular tools. To address this, we employed SRiApt, an aptamer generated through the recently established Blocker‐SELEX pipeline. Its biological stability is improved by phosphorothioate modifications to form PT_f_‐SRiApt. Using this aptamer, the critical role of the SCAF4‐POLR2A interaction in driving TNBC tumor growth and immune regulation is uncovered. PT_f_‐SRiApt effectively inhibits tumor growth and induces cell cycle arrest in TNBC cells with elevated SCAF4 and POLR2A expression. Additionally, PT_f_‐SRiApt promotes premature mRNA termination, boosting antigen presentation and promoting T‐cell infiltration. Analysis of patient samples further confirmed the negative correlation between SCAF4‐POLR2A interaction and the effectiveness of immunotherapy, highlighting the potential of PT_f_‐SRiApt in improving immune efficacy. Together, the work provides a powerful tool not only for dissecting previously “undruggable” protein‐protein interactions but also for enhancing tumor immunogenicity and reshaping the tumor microenvironment.

## Graphical abstract

A phosphorothioate‐modified aptamer PT_f_‐SRiApt capable of disrupting “undruggable” transcription factor SCAF4‐POLR2A interaction with high inhibitory activity and biological stability is presented here. Through this inhibition, PT_f_‐SRiApt successfully arrested cell cycle progression, induced cell death, and elevated immune recruitment in SCAF4‐POLR2A‐high triple‐negative breast cancer. This novel inhibitor also demonstrated potential clinical efficacy in combination with immune checkpoint blockade in a wide range of cancers.

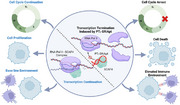

## 1 Introduction

Triple‐negative breast cancer (TNBC) is an aggressive subtype of breast cancer distinguished by the presence of unique super‐enhancers that regulate critical oncogenes involved in tumor progression.^[^ ^1^ ^]^ These enhancer regions are characterized by sustained active transcription and exhibit heightened sensitivity to disruptions in transcriptional activity.^[^ ^2^, ^3^ ^]^ While the targeting of kinases that influence the transcriptional process, such as eEF2K and various cyclin‐dependent kinases that modulate transcription factor complexes,^[^ ^4^, ^5^, ^6^ ^]^ has emerged as a prominent approach to TNBC treatment, there remains a lack of therapeutic strategies that directly target transcription factors (TFs) in this context. One important TF interaction driving transcriptional stability is between SCAF4 and POLR2A.^[^ ^7^, ^8^, ^9^, ^10^ ^]^ This interaction is critical for proper mRNA termination, ensuring the full‐length expression of functional proteins by suppressing early alternative polyadenylation sites, which otherwise produce truncated, non‐functional mRNAs.^[^ ^7^ ^]^ While this interaction's role in normal cellular function is well‐characterized, its contribution to cancer biology remains understudied, largely due to the lack of small‐molecule inhibitors – an issue attributed to the “undruggable” nature of the involved protein surfaces.^[^ ^11^ ^]^

Aptamers, short oligonucleotides that bind targets with high specificity and affinity, represent a powerful alternative approach for modulating protein‐protein interactions.^[^ ^12^ ^]^ Unlike small molecules, aptamers exhibit remarkable versatility in binding a broad range of targets, including proteins, lipids, and metabolites, positioning them as valuable chemical tools in regulating protein‐protein interactions.^[^ ^13^, ^14^ ^]^ Several aptamer‐based therapeutics targeting protein‐protein interactions have achieved significant regulatory milestones. Pegaptanib sodium (Macugen) and avacincaptad pegol (IZERVAY) have received the U.S. Food and Drug Administration (FDA) approval, underscoring their clinical relevance.^[^ ^15^, ^16^ ^]^ Other candidates, such as lexaptepid pegol (NOX‐H94) and BB‐031, are currently in phase II clinical trials.^[^ ^17^, ^18^, ^19^ ^]^ Additionally, the FDA has granted orphan drug designation to Apc001PE.^[^ ^20^ ^]^ Collectively, these advances highlight the clinical viability and growing potential of aptamers as therapeutic agents.

To develop aptamers that can specifically block protein‐protein interactions, we recently reported the Blocker‐SELEX pipeline.^[^ ^21^ ^]^ Among the inhibitory aptamers identified, SRiApt has shown practical promise by effectively inhibiting the interaction between SCAF4/8 and POLR2A. However, the poor biological stability of SRiApt has limited its cellular applicability and hindered a comprehensive evaluation of the therapeutic potential of targeting SCAF4‐POLR2A interactions.

In this study, we first addressed this issue by improving the biological stability of SRiApt through phosphorothioate (PT) modifications.^[^ ^22^, ^23^ ^]^ Leveraging this PT‐modified SRiApt, we demonstrated that it selectively induces cell cycle arrest and impairs the growth of TNBC cell lines characterized by elevated SCAF4‐POLR2A expression. Disruption of SCAF4‐POLR2A interaction with PT_f_‐SRiApt promotes transcription early termination and thus activates the major histocompatibility complex (MHC) class II pathway. Moreover, in vivo assays demonstrated that PT‐modified SRiApt activates the antigen presentation pathway, leading to increased infiltration of T cells. Collectively, our findings reveal the function and therapeutic potential of blocking SCAF4‐POLR2A interaction in TNBC and underscore the utility of PT_f_‐SRiApt as a molecular tool for modulating tumor immunity in TNBC.

## 2 Results and Discussion

### 2.1 PT Modification Improves the Stability and Performance of SRiApt

SRiApt, developed through our Blocker‐SELEX platform, effectively disrupts SCAF4/8‐POLR2A interaction in vitro.^[^ ^21^ ^]^ However, its application is limited by its susceptibility to metabolic degradation. Given the crucial role of chemical modifications in optimizing the drug‐like properties of nucleic acid‐based therapeutics, we sought to improve the stability and efficacy of SRiApt through PT modification, a well‐established approach to improve both stability and affinity of nucleic acid drugs.^[^ ^22^, ^23^ ^]^

**Figure 1:**
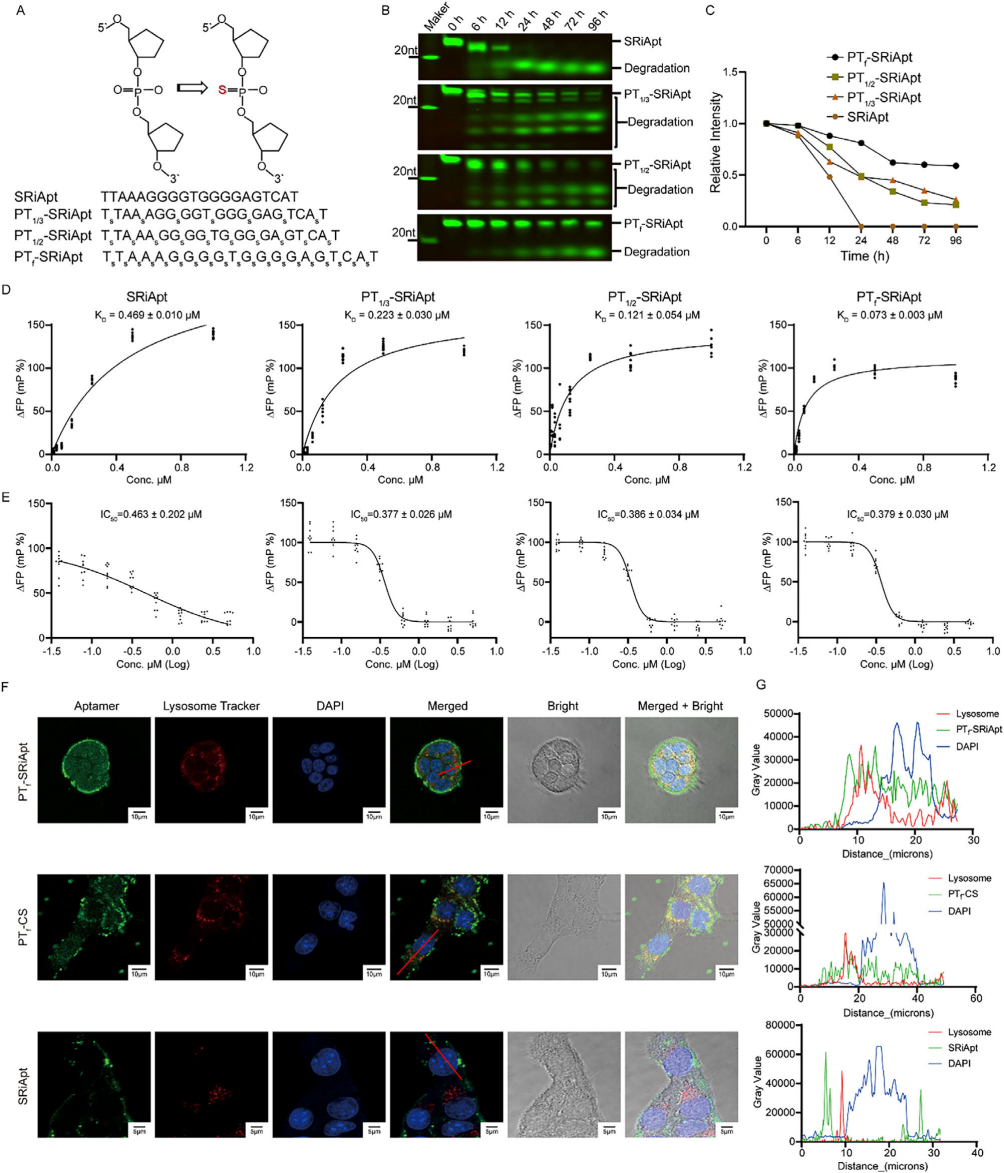
Characterization of the biological stability, binding affinities, and IC_50_ values of PT‐modified SRiApt variants. A) Schematic of SRiApt variants with different degrees of PT modification. B‐C) Evaluation of the metabolic stability of PT‐modified SRiApt variants in serum, showing their susceptibility to degradation. D) Binding affinities of PT‐modified SRiApt variants to SCAF4 protein, as determined using fluorescence polarization (FP) assays. Data represent the mean ± SD (*n* = 3). E) IC_50_ values of PT‐modified SRiApt variants in inhibiting SCAF4‐POLR2A interaction, as measured by competitive FP assays. Data represent the mean ± SD (*n* = 3). F) Confocal microscopy images depicting the endocytosis of PT_f_‐SRiApt, PT_f_‐CS, and SRiApt in 4T1 cells. Cells were treated with 1 µm of corresponding FAM‐labelled oligonucleotide for 24 h at 37 °C, followed by co‐staining with a lysosome tracker and DAPI after formaldehyde fixation. G) Quantification of fluorescence intensities from FAM‐PT_f_‐SRiApt (top), FAM‐PT_f_‐CS (middle), and FAM‐SRiApt (bottom), lysosome tracker, and DAPI in grayscale values following the red line in F).

In this study, we introduced three levels of PT modification to SRiApt: one‐third, one‐half, and full PT substitution (**Figure** **1**A). To evaluate the biological stability of these modified aptamers, we incubated FAM (Fluorescein Amidite)‐labeled SRiApt variants in DMEM with 20% FBS at 37 °C. Electrophoretic analysis and quantification revealed that the fully modified PT_f_‐SRiApt exhibited the highest stability with only minimal degradation observed, while partial modifications (PT_1/3_ and PT_1/2_) resulted in more significant degradation (Figure 1B,C). Next, we evaluated the binding affinities of the SRiApt variants to SCAF4, using fluorescence polarization (FP) assays. The binding affinities (K_D_) were determined to be 0.469±0.010 µm for unmodified SRiApt, 0.223±0.030 µm for PT_1/3_‐SRiApt, 0.121±0.054 µm for PT_1/2_‐SRiApt, and 0.073±0.003 µm for PT_f_‐SRiApt, with the fully modified aptamer showing the strongest binding affinity (Figure 1D). Additionally, PT‐modified variants exhibited comparable IC_50_ values in competitive FP assays for inhibiting SCAF4‐POLR2A interaction against unmodified SRiApt: 0.463±0.202 µm for unmodified SRiApt, 0.377±0.026 µm for PT_1/3_‐SRiApt, 0.386±0.034 µm for PT_1/2_‐SRiApt, and 0.379±0.030 µm for PT_f_‐SRiApt (Figure 1E). Noticeably, PT modification resulted in a steeper IC50 binding curve, implying increased binding affinity or the stabilization of binding‐competent conformations.^[^ ^23^, ^24^, ^25^ ^]^

These findings demonstrate that PT_f_‐SRiApt exhibits the highest binding affinity for SCAF4 with comparable IC_50_ values across variants. Given these improved characteristics, PT_f_‐SRiApt was selected for further cellular intake studies using three cell lines (4T1, AGS, MCF1A). When FAM‐labeled PT_f_‐SRiApt (1 µm) was introduced to the culture medium, it was efficiently internalized by all cells tested within 24 h (Figure 1F; Figure S1A, Supporting Information). To determine the effects of PT modification on the intracellular localization of SRiApt, we performed colocalization studies using a lysosome tracker and DAPI nuclear stain. The results revealed that FAM‐labeled PT_f_‐SRiApt was evenly distributed throughout the cytoplasm with no detectable accumulation in lysosomes, indicating effective cellular delivery without lysosomal degradation (Figure 1G; Figure S1B, Supporting Information). In contrast, the unmodified aptamer showed no detectable cellular uptake, highlighting the importance of chemical modification for membrane permeability. Interestingly, the modified control sequence also exhibited cellular uptake comparable to PT_f_‐SRiApt, suggesting that the observed internalization was determined by PT modifications (Figure 1G; Figure S1B, Supporting Information). This phenomenon was coherently observed across all three cell lines tested, indicating PT modification's consistent and broad enhancement of cellular uptake efficiency, in alignment with previously reported findings in the literature.^[^ ^26^ ^]^ In summary, PT modification significantly augments SRiApt, improving its biological stability, binding affinity, and cellular uptake, reinforcing its potential as a modulating agent for inhibiting SCAF4/8‐POLR2A interactions.

### 2.2 PT_f_‐SRiApt Selectively Impairs the Growth of TNBC Cancer Cells with Elevated SCAF4 and POLR2A Expression Levels

**Figure 2:**
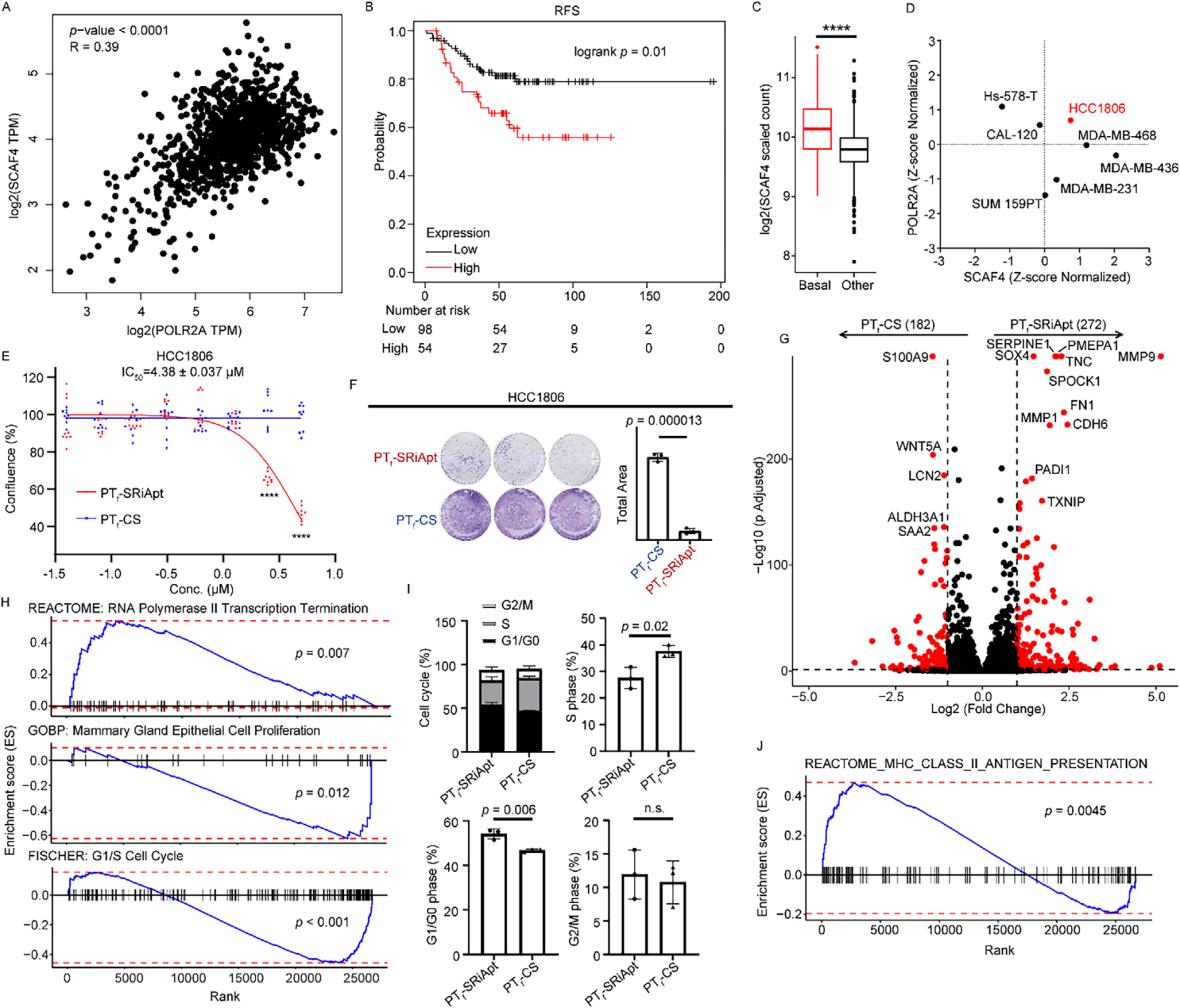
Biological effects of PT_f_‐SRiApt on arresting breast cancer progression. A) Spearman correlation analysis of SCAF4 and POLR2A expression levels in breast cancer samples, as analyzed using GEPIA. B) Kaplan‐Meier survival analysis showing reduced relapse‐free survival (RFS) in breast cancer patients with high co‐expression of SCAF4 and POLR2A. C) Comparison of SCAF4 expression in TNBC versus other subtypes from The Cancer Genome Atlas (TCGA) dataset; **** indicates *p* < 0.0001, determined using two‐tailed Wilcoxon test. D) Expression profiles of SCAF4 and POLR2A across seven human TNBC cell lines. E) Growth curves of HCC1806 cells treated with PT_f_‐SRiApt or PT_f_‐CS for five days (*n* = 3). Data are presented as mean ± SD; **** indicates *p* < 0.0001, determined using a two‐tailed Student's t‐test. F) Anti‐tumorigenic effects of PT_f_‐SRiApt on HCC1806 cells, as determined by crystal violet staining assays (*n* = 3). *p*‐value determined by two‐tailed Student's t‐test. G) Volcano plot displaying the log_2_ fold change of significantly upregulated and downregulated genes in 4T1 cells treated with PT_f_‐SRiApt. Genes with significant changes were shown in red (*p* < 0.05, Log_2_ (fold change) > 1). H) GSEA analysis of pathways regulated by PT_f_‐SRiApt, showing significant activation of the RNA Polymerase II Transcription Termination pathway (*p* = 0.007) and significant downregulation of the Mammary Gland Epithelial Cell Proliferation (*p* = 0.012) and G1/S Cell Cycle pathways (*p* < 0.001). The *p*‐value was determined using a permutation test. I) Flow cytometric analysis of cell cycle distribution in 4T1 cells treated with 1 µm PT_f_‐SRiApt or PT_f_‐CS. Data are presented as mean ± SD with *p*‐values calculated using a two‐tailed Student's t‐test. n.s. Indicates not significant. J) GSEA of the REACTOME_MHC_Class_II_antigen_presentation pathway, significantly upregulated following PT_f_‐SRiApt treatment (*p* = 0.0045). The *p*‐value was determined using a permutation test.

The interaction between SCAF4 and POLR2A is integral to maintaining transcriptional fidelity, yet its role in cancer remains unexplored.^[^ ^8^ ^]^ Through Gene Expression Profiling Interactive Analysis (GEPIA)^[^ ^27^ ^]^ of breast cancer datasets, we uncovered a strong positive correlation between SCAF4 and POLR2A gene expression across multiple breast cancer subtypes (**Figure** **2**A). Survival analysis further revealed that co‐expression of these genes is significantly associated with poorer clinical outcomes, including reduced relapse‐free survival (RFS) and diminished distant metastasis‐free (DMFS) survival (*p* = 0.036), as analyzed by Kaplan‐Meier Plotter,^[^ ^28^ ^]^ highlighting the clinical relevance of this interaction in breast cancer progression (Figure 2B; Figure S2, Supporting Information). Further analysis indicated that TNBC samples exhibited significantly higher SCAF4 expression compared to other breast cancer subtypes (Figure 2C). This suggests that SCAF4 may play a pivotal role in transcription regulation and stability in TNBC, making the SCAF4‐POLR2A interaction a compelling target for therapeutic intervention in this particularly aggressive cancer subtype.

To evaluate the antiproliferative effects of PT_f_‐SRiApt, we conducted a proliferation screening assay across seven human‐derived TNBC cancer cell lines. PT_f_‐SRiApt exhibited potent inhibitory activity, particularly in the HCC1806 cell line, while PT_f_‐CS, a fully PT‐modified control sequence, had no significant effect (Figure S3, Supporting Information). Notably, HCC1806 cells, which were the most sensitive to PT_f_‐SRiApt (IC_50_ = 4.38 ± 0.04 µm), exhibited elevated co‐expression of SCAF4 and POLR2A (Figure 2D,E). In contrast, cell lines with discordant expressions of these genes showed reduced sensitivity to treatment (Figure S3, Supporting Information). This trend was similarly observed in gastric cancer cell lines AGS and HGC27, further supporting the specificity of PT_f_‐SRiApt's anti‐proliferative activity (Figure S4, Supporting Information). These data suggest that PT_f_‐SRiApt impairs cell proliferation by specifically targeting SCAF4‐POLR2A interaction, with therapeutic activity depending on the high expression of these two genes. In addition to its anti‐proliferative effects, PT_f_‐SRiApt markedly inhibited long‐term tumorigenic potential in HCC1806 cells compared to PT_f_‐CS control, as demonstrated by colony formation assays (Figure 2F). This dual impact on proliferation and tumorigenesis strengthens the antitumor activity of PT_f_‐SRiApt for TNBC with high SCAF4‐POLR2A expression.

To explore the underlying molecular mechanisms, we conducted bulk RNA sequencing on PT_f_‐SRiApt‐treated HCC1806 cells. Compared to cells treated with the control sequence, the transcriptomic analysis revealed significant changes in gene expression, with 272 up‐regulated and 182 downregulated genes up‐on treatment (*p* < 0.05, Log_2_ (fold change) > 1) (Figure 2G). Gene set enrichment analysis (GSEA) identified activation of the RNA Polymerase II Transcription Termination pathway (*p* = 0.007), suggesting the on‐target effect of PT_f_‐SRiApt promotes premature transcription termination by disrupting the SCAF4‐POLR2A interaction (Figure 2H). Concurrently, the Mammary Gland Epithelial Cell Proliferation pathway was significantly downregulated (*p* = 0.012), correlating with the observed inhibition of cell proliferation (Figure 2H). Further pathway analysis highlighted the downregulation of G1/S cell cycle transition genes, implicating PT_f_‐SRiApt in inducing cell cycle arrest at the G0/G1 phase (Figure 2H). Flow cytometry corroborated this finding, showing a marked increase in G0/G1 phase cell population and a corresponding decrease in S phase cell population (Figure 2I; Figure S5, Supporting Information), further supporting the antiproliferative activity of PT_f_‐SRiApt.

Interestingly, PT_f_‐SRiApt treatment also led to the enrichment of the MHC class II antigen presentation pathway (Figure 2J). This suggests that polypeptides derived from prematurely terminated transcripts, resulting from the disruption of SCAF4‐POLR2A interaction, may undergo rapid proteasomal degradation, thereby promoting neoantigen generation and subsequent MHC‐mediated presentation.^[^ ^29^ ^]^ The improved antigen presentation may represent an additional mechanism contributing to the antitumor efficacy of PT_f_‐SRiApt, suggesting that it could promote immune‐mediated tumor suppression beyond its direct inhibitory effects on proliferation.

### 2.3 PT_f_‐SRiApt Suppresses Tumor Growth In Vivo

**Figure 3:**
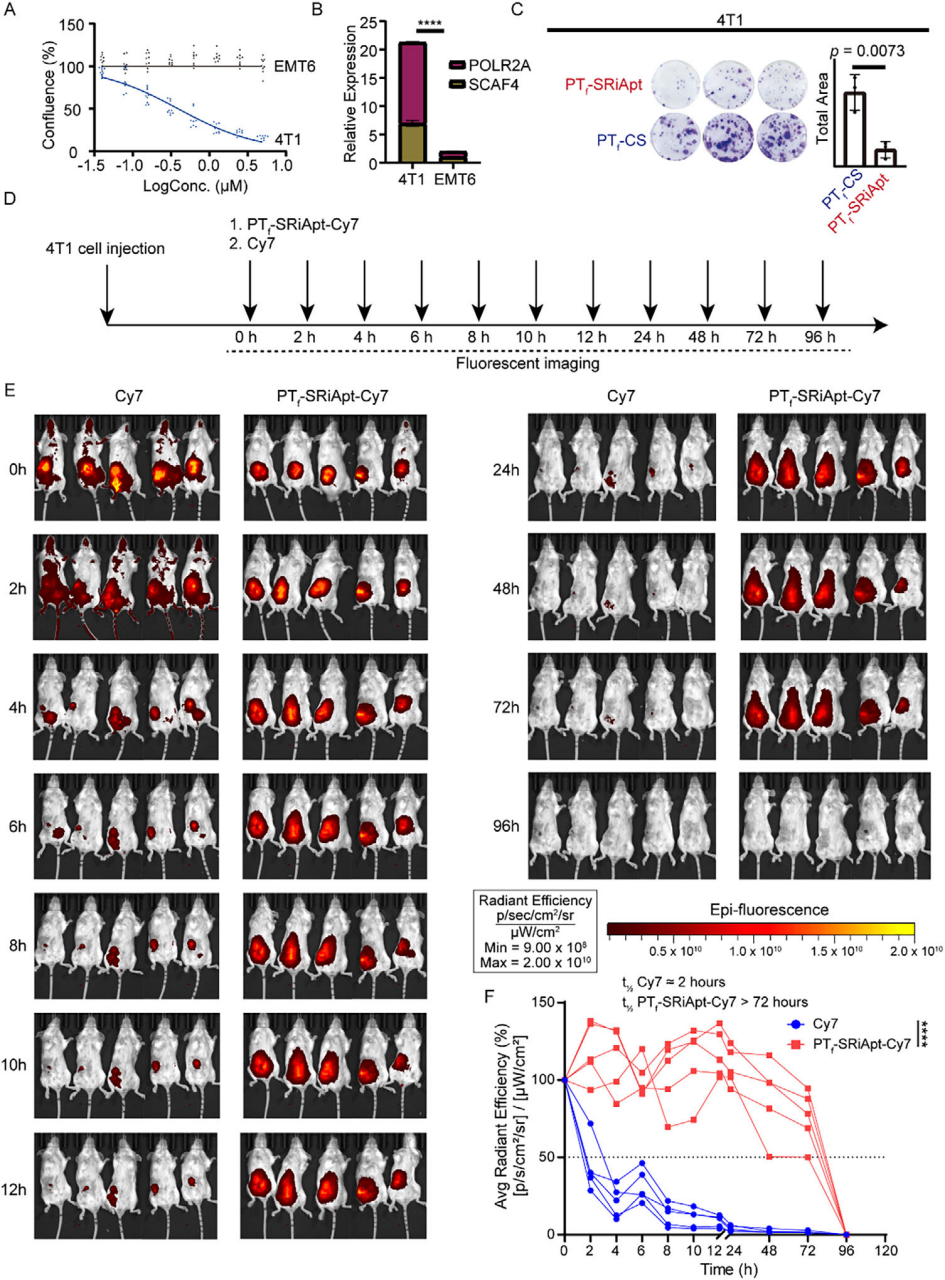
Pharmacokinetic characterization of PT_f_‐SRiApt in murine 4T1 breast cancer model. A) Growth curves of 4T1 and EMT6 cells treated with PT_f_‐SRiApt over 5 days. B) Relative expression levels of SCAF4 and POLR2A in 4T1 and EMT6 cell lines. The *p*‐value was determined using a two‐way ANOVA test. C) Anti‐tumorigenesis effects of PT_f_‐SRiApt or PT_f_‐CS on 4T1 cells, as assessed via crystal violet staining assay (*n* = 3). The *p*‐value was determined by a two‐tailed Student's t‐test. D) Schematic illustration of the fluorescent measurement schedule of mice injected with PT_f_‐SRiApt‐Cy7 (*n* = 5) and Cy7 (*n* = 5). E) Epi‐fluorescence images showing the in vivo distribution of free Cy7 dye versus PT_f_‐SRiApt‐Cy7 in mice over a 96‐h period post‐injection. F) Quantification of fluorescence intensity from panel E over a 96‐h period. The line plot depicts the fluorescence signal of each mouse for free Cy7 and PT_f_‐SRiApt‐Cy7. The *p*‐value was determined by a two‐tailed Student's t‐test. ****, *p* < 0.0001.

To evaluate the therapeutic potential of PT_f_‐SRiApt in vivo, we first investigated its cytotoxic effects on murine‐derived TNBC cell lines. PT_f_‐SRiApt exhibited pronounced cytotoxicity toward 4T1 cells with an IC_50_ of 0.336 ± 0.070 µm, while EMT6 cells displayed limited response (**Figure** **3**A). This differential sensitivity aligns with elevated expression levels of SCAF4 and POLR2A in 4T1 cells, supporting our earlier observations that high SCAF4‐POLR2A co‐expression enhances responsiveness to PT_f_‐SRiApt (Figure 3B). PT_f_‐SRiApt significantly inhibited tumorigenesis in 4T1 cells, as demonstrated by a colony formation assay, further supporting its potential for in vivo applications (Figure 3C).

To evaluate the in vivo behavior of PT_f_‐SRiApt, we first assessed its pharmacokinetics in a 4T1 tumor‐bearing BALB/c mouse model. Once tumors were palpable, mice received intratumoral injections of either free Cy7 dye or PT_f_‐SRiApt conjugated with Cy7, followed by longitudinal epi‐fluorescence imaging over 96 h (Figure 3D). Compared to free Cy7, which exhibited a rapid clearance with a half‐life of ≈2 h, PT_f_‐SRiApt‐Cy7 remained confined to the tumor site with no detectable systemic distribution and demonstrated significantly prolonged intratumoral retention, with a half‐life exceeding 72 h and visible clearance by 96 h (Figure 3E,F). This clearance profile suggests effective metabolism without long‐term accumulation, mitigating the risk of chronic toxicity. Overall, these results indicate that PT_f_‐SRiApt achieves localized and sustained tumor exposure following intratumoral administration, with a favorable safety profile.

**Figure 4:**
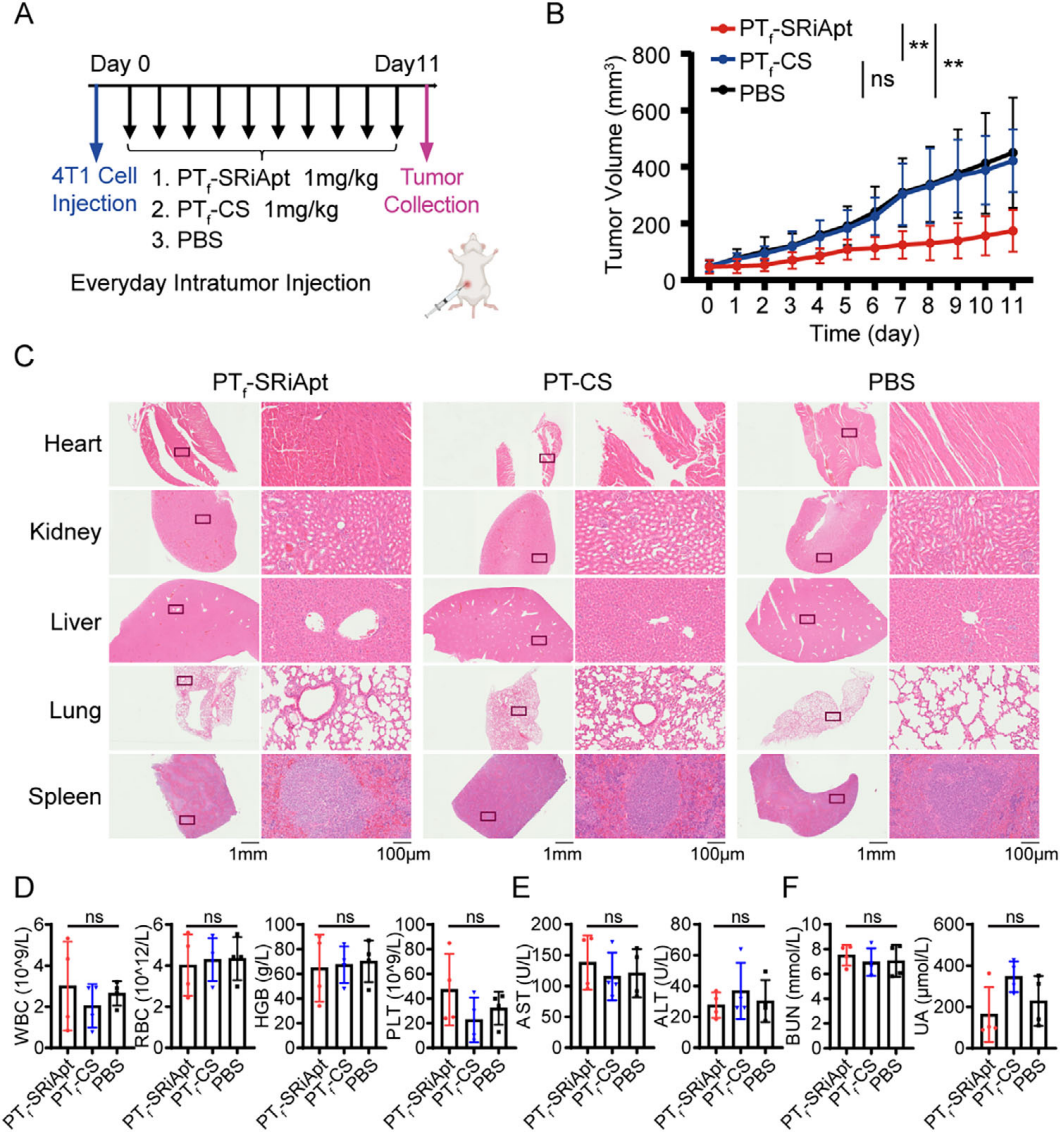
Antitumor efficacy of PT_f_‐SRiApt in murine 4T1 breast cancer model. A) Schematic illustration of the treatment schedule with PT_f_‐SRiApt, PT_f_‐CS, and PBS in the in vivo mouse experiments. B) Averaged tumor volume curves of mice treated with PBS (black, *n* = 7), PT_f_‐CS (blue, *n* = 7), and PT_f_‐SRiApt (red, *n* = 8). Data represent mean ± SD of tumor volumes calculated from the diameter of the tumor mass. Differences at day 11 were considered statistically significant if the *p*‐value < 0.05, determined by one‐way ANOVA with Tukey's multiple comparisons test. **, *p* < 0.01; ns, not significant. C) H&E imaging with zoomed regions (black box) of the main organs (heart, liver, kidney, lung, and spleen) treated with PT_f_‐SriApt (*n* = 4), PT_f_‐CS (*n* = 4), or PBS (*n* = 4) in mice. D) Complete blood count of 4T1 bearing BALB/c mice after PT_f_‐SRiApt (*n* = 4), PT_f_‐CS (*n* = 4), or PBS (n = 4) treatment (WBC: white blood cell, RBC: red blood cell, HGB: hemoglobin, PLT: platelet). E) Liver function test of 4T1 bearing BALB/c mice after PT_f_‐SRiApt (*n* = 4), PT_f_‐CS (*n* = 4), or PBS (*n* = 4) treatment (AST: aspartate transaminase, ALT: alanine transaminase). F) Kidney function test of 4T1 bearing BALB/c mice after PT_f_‐SRiApt (*n* = 4), PT_f_‐CS (*n* = 4), or PBS (*n* = 4) treatment (BUN: blood urea nitrogen, UA: uric acid). Significance was determined using a one‐way ANOVA test. ns, not significant.

Upon establishing the pharmacokinetic properties of PT_f_‐SRiApt, we first investigated its efficacy in vivo employing a 4T1 tumor‐bearing model in immunodeficient nude mice. Once the tumors became palpable, mice were administered daily with intratumoral injections of 1 µg kg^−1^ of PT_f_‐SRiApt or PT_f_‐CS (Figure S6A, Supporting Information). Tumor volumes were measured daily and calculated using the standard formula. PT_f_‐SRiApt significantly inhibited tumor growth compared to PT_f_‐CS control (Figure S6B, Supporting Information). Body weight monitoring revealed no detectable systemic toxicity at this dosage (Figure S6C, Supporting Information). We next tested PT_f_‐SRiApt in an immunocompetent BALB/c mouse model to further explore its therapeutic potential. Mice were grouped into three cohorts, including PT_f_‐SRiApt, PT_f_‐CS, and PBS, and treated with 1 µg kg^−1^ of PT_f_‐SRiApt, PT_f_‐CS, or PBS administered daily via intratumoral injection once the tumors became palpable (**Figure** **4**A). Remarkably, PT_f_‐SRiApt significantly suppressed tumor growth compared to PT_f_‐CS and PBS with no observable body weight loss or organ toxicity through H&E histological staining of heart, kidney, liver, lung, and spleen (Figure 4B,C, Figures S7 and S8, Supporting Information). Furthermore, no alterations to complete blood count, liver function, and kidney function test results were visible after PT_f_‐SRiApt treatment, suggesting a lack of toxicity (Figure 4D–F). Importantly, the absence of significant immune response in the PT_f_‐CS group suggests that the antitumor activity of PT_f_‐SRiApt is specifically mediated through targeted disruption of SCAF4‐POLR2A interaction, rather than through nonspecific immune activation by exogenous DNA.

### 2.4 Single‐cell Characterization of PT_f_‐SRiApt's Antitumor Effect

**Figure 5:**
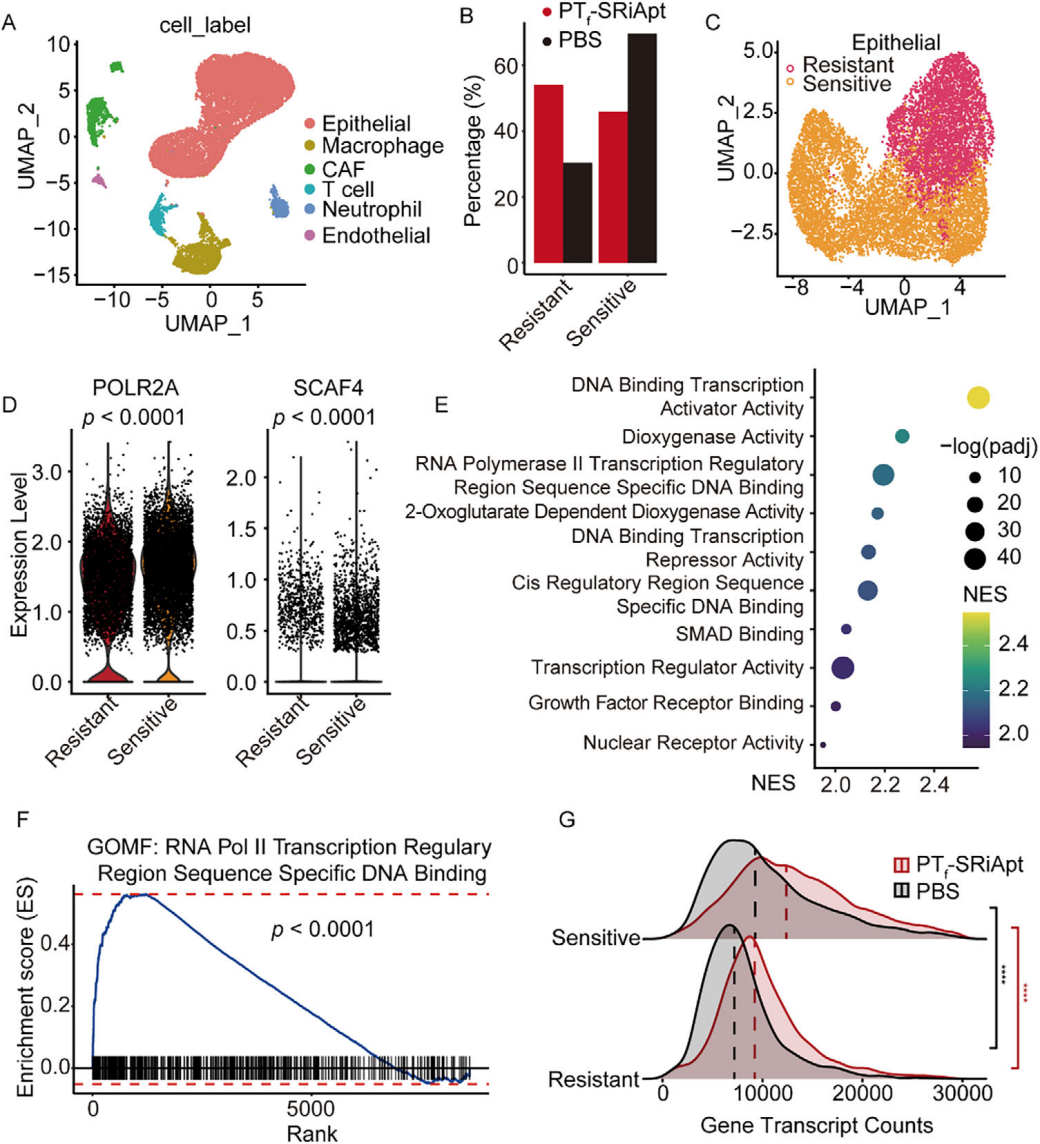
Antitumor efficacy of PT_f_‐SRiApt in murine 4T1 breast cancer model. A) UMAP plot showing cell populations identified within tumors from both PT_f_‐SRiApt and PT_f_‐CS groups. Cell types are labeled on the right. B) Proportional changes of tumor cells in resistant and sensitive subgroups upon PT_f_‐SRiApt treatment. C) UMAP plot of subclustering of 4T1 cell‐derived tumors to resistant and sensitive groups. D) Expression levels of POLR2A and SCAF4 in resistant and sensitive subgroups. *p*‐values were determined by a two‐tailed Wilcoxon test. E) Enrichment analysis of GO terms (BP) in 4T1 cells treated with PT_f_‐SRiApt for 5 days. F) GSEA analysis of the RNA Pol II transcription regulatory pathway regulated by PT_f_‐SRiApt treatment with *p* = 1.73e‐21. G) Density plot of gene counts per cell for each experimental group across sensitive and resistant clusters. Vertical dashed lines indicate median gene counts within each group. The *p*‐values were determined using the Wilcoxon rank sum test. ****, *p* < 0.0001.

To elucidate the underlying mechanism of PT_f_‐SRiApt's antitumor activity, we performed single‐cell RNA sequencing on tumors harvested from PT_f_‐SRiApt treated and control mice after 10 days of treatment. Post‐quality control, we analyzed 17482 high‐quality cells and categorized them into six major cell types based on canonical markers (**Figure** **5**A; Figure S9, Supporting Information). PT_f_‐SRiApt treatment significantly reduced tumor cell proliferation and altered the cellular composition of tumors, as evidenced by a decreased proportion of tumor cells and an increased proportion of macrophages, cancer‐associated fibroblasts (CAFs), T cells, and neutrophils (Figure S10, Supporting Information).

To further investigate the heterogeneity of tumor cells, we divided tumor cells into seven distinct subclusters based on their expression profiles (Figure S11A, Supporting Information). Comparison of these subclusters before and after PT_f_‐SRiApt treatment revealed a compositional change in tumor cells post‐treatment: clusters 0, 3, and 6 increased, while clusters 1, 2, 4, and 5 decreased (Figure S11B, Supporting Information). This differential response suggests varying sensitivity to PT_f_‐SRiApt among the clusters. To explore the basis of this differential sensitivity, we grouped the proportionally increased clusters (0, 3, and 6) into a resistant group and the proportionally decreased clusters (1, 2, 4, and 5) into a sensitive group (Figure 5B). UMAP visualization demonstrated spatial similarities within clusters of the same subgroup and distinct separation between the subgroups suggesting different expression profiles across sensitive and resistant groups (Figure 5C). Expression analysis showed that the sensitive group exhibited significantly higher POLR2A and SCAF4 expression levels compared to the resistant group, providing evidence of PT_f_‐SRiApt's specificity at the single‐cell level (Figure 5D). Pathway enrichment analysis revealed that the resistant group was enriched in transcriptional regulatory pathways, particularly in RNA polymerase II‐related transcription regulatory pathway (Figure 5E,F; Figure S11C, Supporting Information). Moreover, the total transcription level was downregulated in the resistant group as evidenced by the lower average number of transcripts detected per cell compared to the sensitive group (Figure 5G). Higher transcriptional activity in the sensitive group suggested “transcriptionally addicted” cancer cells are more vulnerable to transcriptional dysregulation, a finding that is further supported by existing literature.^[^ ^30^ ^]^ These findings shed light on the mechanistic basis of PT_f_‐SRiApt's selective anti‐proliferative effects on tumor cells in a SCAF4‐POLR2A interaction‐dependent manner.

### 2.5 PT_f_‐SRiApt Promotes T‐Cell Infiltration and Modulates Tumor Microenvironment in the 4T1 Mouse Model

**Figure 6:**
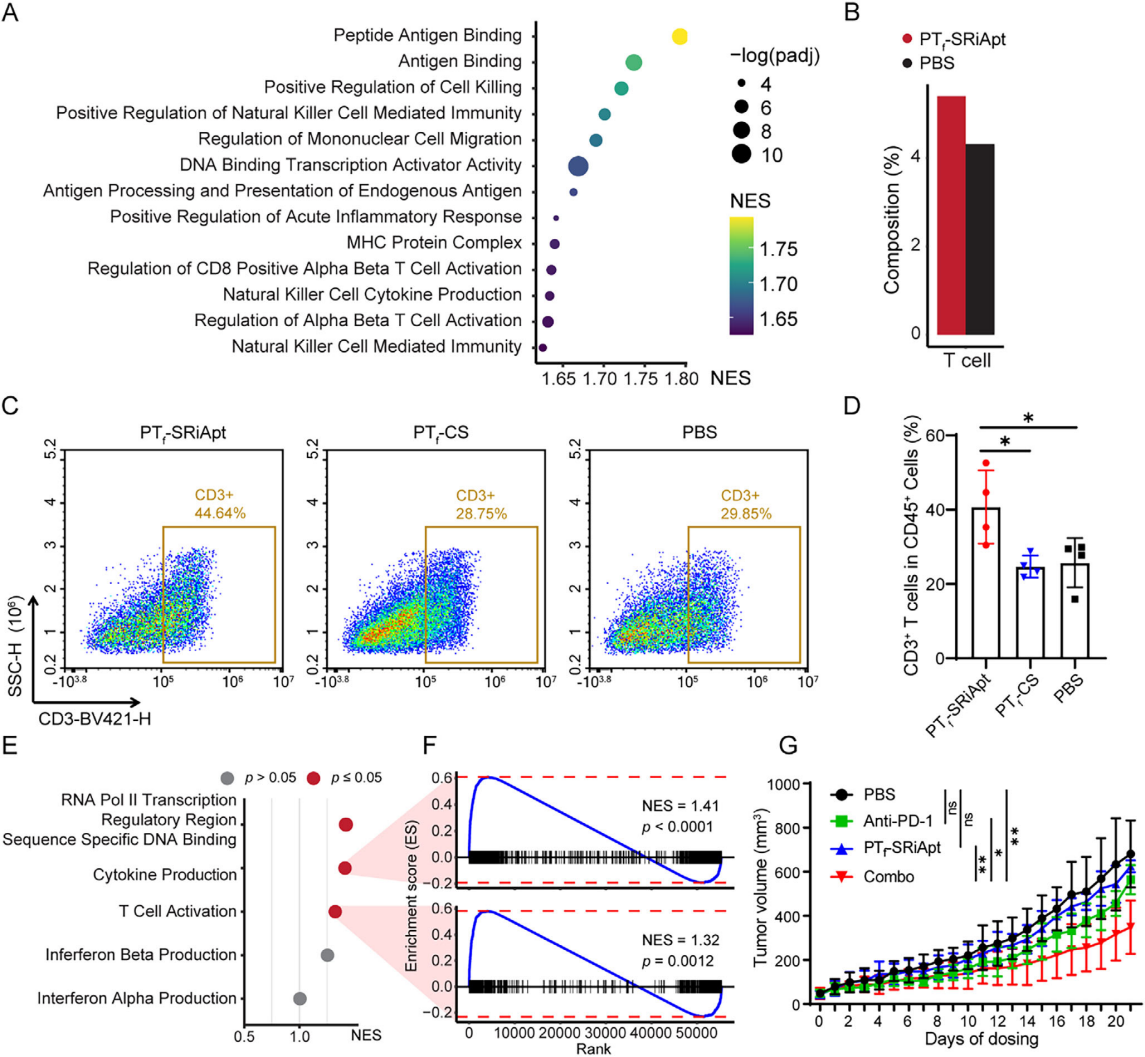
PT_f_‐SRiApt improves T cell populations in breast cancer. A) GSEA enrichment of GO terms in PT_f_‐SRiApt tumor cells versus control PBS tumor cells. B) Proportion of T cells in tumors treated with PT_f_‐SRiApt or PBS. C) Representative flow cytometric analysis of T cells in tumors treated by PT_f_‐SRiApt, PT_f_‐CS, or PBS. D) Populations of T cells in total tumor cells. Statistical analysis from four biological replicates. ***, *p* < 0.001; ns, not significant, as determined by one‐way ANOVA with Tukey's multiple comparisons test. E) GSEA enrichment of GO terms in PT_f_‐SRiApt T cells versus control PBS T cells. F) GSEA plot showing the enrichment of cytokine production and T cell activation pathways in T cells of PT_f_‐SRiApt treated mice. The *p*‐value was determined using a permutation test. G) Tumor growth curves of 4T1 tumor‐bearing mice treated with PBS (*n* = 4), SRiApt (*n* = 5), anti‐PD‐1 (*n* = 4), or a combination of SRiApt and anti‐PD‐1 (*n* = 5). Data represent mean ± SD of tumor volumes. *, *p* < 0.05; **, *p* < 0.01; and ***, *p* < 0.001, determined using unpaired t‐test at day 21.

To determine the phenotype of mice tumor cells post PT_f_‐SRiApt treatment at the single‐cell level, we performed GSEA analysis comparing PT_f_‐SRiApt treated with PBS‐treated control tumor cells. Consistent with bulk RNA sequencing results, antigen binding is one of the most enriched pathways in PT_f_‐SRiApt treated tumor cells (**Figure** **6**A). Additionally, enrichment in the MHC complex, activation of alpha‐beta T cell, and mononuclear cell migration suggest PT_f_‐SRiApt treatment induces antigen binding and presentation in tumor cells, which in turn recruits and activates leukocytes. This observation was further supported by notable alterations in the cellular composition of 4T1 tumors treated with PT_f_‐SRiApt, particularly a pronounced increase in T cell infiltration (Figure 6B, Figure S10, Supporting Information). Flow cytometry analysis was conducted to experimentally validate this finding, revealing a substantial enrichment of T cells in PT_f_‐SRiApt‐treated tumors compared to PT_f_‐CS and PBS controls (Figure 6C,D, Figure S12, Supporting Information). Additionally, a comparison of PT_f_‐SRiApt's therapeutic efficacy in immunocompetent versus immunodeficient mice revealed significantly better tumor suppression in immunocompetent mice (*p* = 0.0109), further underscoring the role of immune activation in PT_f_‐SRiApt's antitumor activity (Figure S13, Supporting Information).

To investigate the mechanism by which PT_f_‐SRiApt enhances T cell infiltration into the tumor microenvironment, we first examined whether this effect could be attributed to direct modulation of T cell activity. In vitro assays using CCK8 for proliferation and flow cytometric analysis for activation marker CD69 revealed that PT_f_‐SRiApt alone does not directly influence T cell proliferation or activation (Figure S14A,B, Supporting Information). Furthermore, co‐culture experiments comparing tumor cells treated with PT_f_‐SRiApt alone versus those co‐cultured with both PT_f_‐SRiApt and T cells showed no significant difference in tumor cell killing, indicating that PT_f_‐SRiApt does not directly induce T cell‐mediated cytotoxicity under these conditions (Figure S14C, Supporting Information). In contrast, single‐cell RNA sequencing of tumors from PT_f_‐SRiApt–treated mice revealed transcriptional signatures consistent with increased T cell activation within the tumor microenvironment (Figure 6E,F). These results align with the bulk and single‐cell transcriptomic analyses, which revealed upregulation of MHC and antigen presentation pathways in tumor cells following PT_f_‐SRiApt treatment (Figures 2J and 6A), implicating enhanced antigen presentation as a mechanism underlying the observed immunomodulatory effects.

Given that enhanced immune infiltration and activation are often associated with improved responsiveness to immune checkpoint blockade (ICB),^[^ ^31^, ^32^ ^]^ we next evaluated whether PT_f_‐SRiApt could potentiate immunotherapeutic efficacy. In the 4T1 syngeneic tumor model, treatment with either a reduced dose of PT_f_‐SRiApt (0.5 mg kg^−1^) or anti‐PD‐1 antibody (200 µg) alone had no significant effect on tumor growth. However, combination therapy with a reduced dose of PT_f_‐SRiApt and anti‐PD‐1 led to a significant reduction in tumor volume (Figure 6G; Figure S15, Supporting Information). These results suggest that PT_f_‐SRiApt synergizes with ICB therapy, likely by reshaping the tumor immune landscape to favor increased T cell activation and infiltration.

### 2.6 SCAF4‐POLR2A Interaction Predicts Poor Tumor Immunity in TNBC Patients

**Figure 7:**
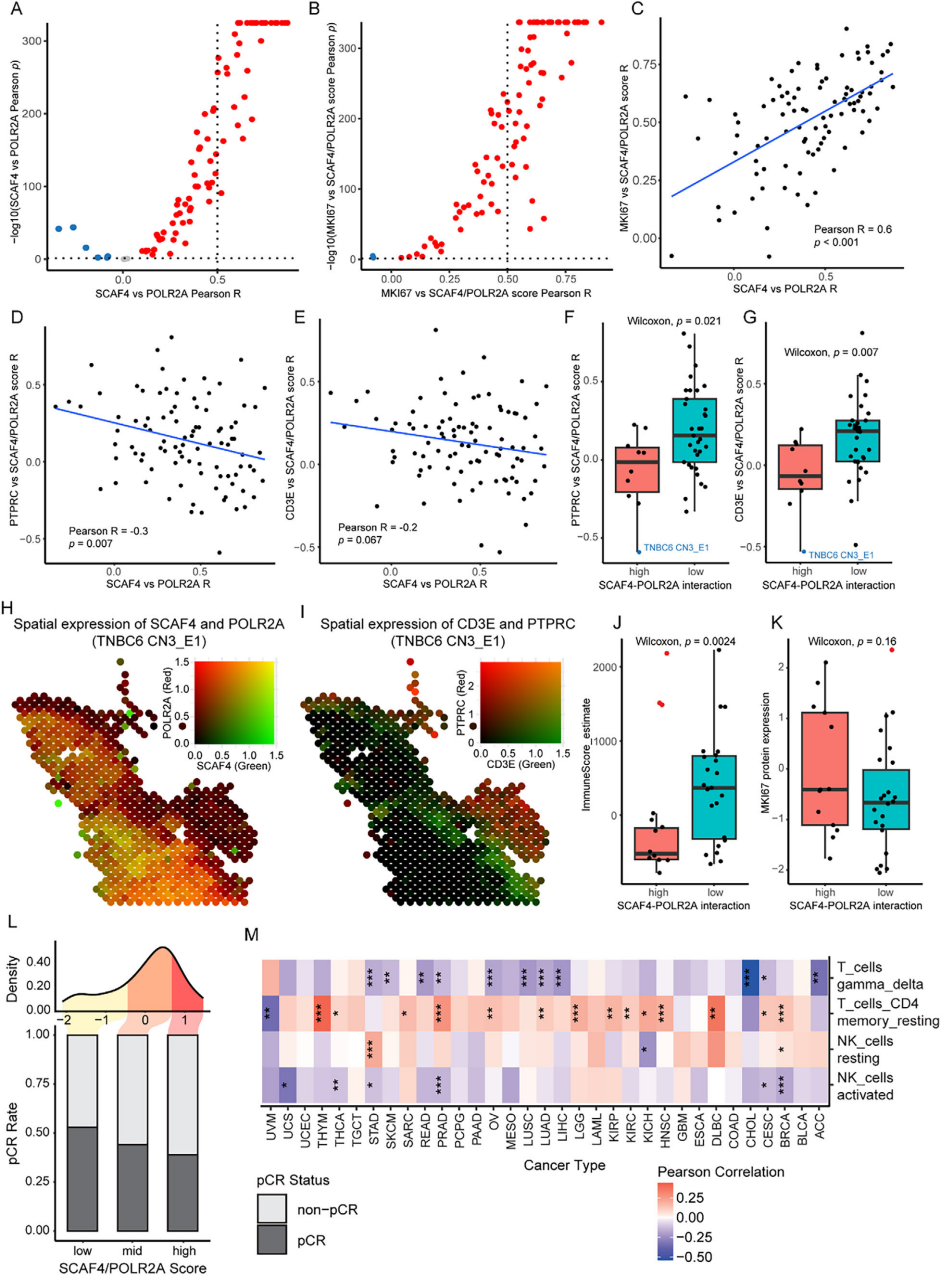
SCAF4‐POLR2A interaction promotes tumor proliferation and inhibits immune infiltration in TNBC patients. A) Per bin Pearson correlation coefficient and significance between SCAF4 and POLR2A expression in TNBC ST samples (*n* = 94). B) Per bin Pearson correlation coefficient and significance between SCAF4‐POLR2A score and MKI67 expression in TNBC ST samples (*n* = 94). C‐E) Pearson correlation between (C) MKI67/SCAF4‐POLR2A, (D) PTPRC/SCAF4‐POLR2A and (E) CD3E/SCAF4‐POLR2A Pearson coefficient with SCAF4/POLR2A Pearson coefficient (*n* = 94). F) Comparison between ESTIMATE immune score in SCAF4‐POLR2A high and low interaction ST samples (*n* = 94). G) Comparison between MKI67 expression in SCAF4‐POLR2A high and low interaction ST samples (*n* = 94). H) Spatial visualization of SCAF4 and POLR2A co‐expression. I) Spatial visualization of CD3E and PTPRC co‐expression. J) Comparison between ESTIMATE immune score in SCAF4‐POLR2A high and low interaction proteomic samples (*n* = 55). K) Comparison between MKI67 expression in SCAF4‐POLR2A high and low interaction proteomic samples (*n* = 55). Panels F, G, J, K: *p*‐values determined using two‐tailed Wilcoxon test. L) pCR rate of pembrolizumab treatment in patients grouped by SCAF4‐POLR2A expression in the I‐SPY2 clinical trial. G) Association of SCAF4‐POLR2A expression with T cells and NK cells deconvolution across TCGA cancer types. *, *p* < 0.05; **, *p* < 0.01; and ***, *p* < 0.001, determined using asymptotic confidence intervals based on Fisher transformation.

To explore the clinical relevance of targeting the SCAF4–POLR2A interaction, we next sought to determine whether its expression is associated with immune activity in human tumors. Leveraging publicly available TNBC spatial transcriptomic (ST) data, we conducted a comprehensive analysis of 94 publicly available samples to explore the spatial correlation between SCAF4‐POLR2A interaction and tumor immunity.^[^ ^33^ ^]^ After imputing dropout values, we calculated the Pearson correlation between SCAF4 and POLR2A expression across spatial transcriptomic spots. The majority of samples (83 out of 94) showed a statistically significant positive correlation, with 38 displaying strong spatial co‐expression (Pearson R > 0.5), indicating that SCAF4 and POLR2A are frequently co‐expressed in the tumor microenvironment (**Figure** **7**A).

To determine whether this spatial interaction reflects changes in tumor proliferation and immunity, we first calculated SCAF4‐POLR2A co‐expression by averaging the normalized expression of both genes at each spot. We then assessed the spatial correlation between the SCAF4‐POLR2A co‐expression and the proliferation marker MKI67, which revealed that most samples displayed a strong positive correlation, suggesting that regions with high SCAF4–POLR2A co‐expression are associated with increased tumor proliferation (Figure 7B). Further analysis revealed that samples with high SCAF4‐POLR2A co‐localization also exhibit a strong spatial correlation between MKI67 expression and SCAF4‐POLR2A co‐expression (Figure 7C). In contrast, these same samples show a low spatial correlation between immune markers (PTPRC and CD3E) and SCAF4‐POLR2A co‐expression (Figure 7D,E). Together, these results suggest that strong SCAF4‐POLR2A co‐expression is localized with tumor progression and linked to spatial exclusion of immune infiltration.

To further illustrate this trend, we stratified samples into high‐ and low‐interaction groups based on pseudobulk expression profiles. Tumors with above‐average expression of both SCAF4 and POLR2A were defined as high‐interaction, while those with below‐average expression of both genes were classified as low‐interaction. High‐interaction tumors showed more negative spatial correlations between immune markers and the interaction score, further supporting the association between SCAF4–POLR2A activity and immune exclusion (Figure 7F,G). Representative spatial plots further illustrate this dissociation (e.g., TNBC6 CN3_E1; Figure 7H,I).

Complementing these findings at the protein level, analysis on a public TNBC proteomic dataset by Anurag et al.^[^ ^34^ ^]^ showed that high SCAF4–POLR2A interaction was associated with significantly lower immune scores based on ESTIMATE deconvolution, supporting the negative relationship between this interaction and immune infiltration (Figure 7J). Additionally, while MKI67 expression trended higher in the high‐interaction group, this difference was not statistically significant (*p* = 0.16; Figure 7K), but remains consistent with the transcriptomic data. These results suggest that in TNBC clinical samples, SCAF4–POLR2A co‐expression is linked to tumor proliferation and immune exclusion, reinforcing the potential of targeting this axis to improve immunotherapeutic outcomes.

The pembrolizumab (Keytruda, anti‐PD‐1 antibody) treatment arm of the I‐SPY2 clinical trial was then analyzed to examine the effect of SCAF4‐POLR2A interaction in immunotherapy.^[^ ^35^ ^]^ Patients with high expression levels of both genes exhibited poor response rates to pembrolizumab, whereas patients with moderate expression levels showed intermediate responsiveness, and those with low expression experienced the most favorable outcomes. This suggests that SCAF4 and POLR2A expressions could serve as predictive biomarkers for immunotherapy response, positioning PT_f_‐SRiApt as a potential booster of immunotherapy efficacy by disrupting SCAF4‐POLR2A interaction (Figure 7L).

SCAF4‐POLR2A expression also correlates to immune activation in pan‐cancer. Using Cancer Genome Atlas (TCGA) data,^[^ ^36^ ^]^ we observed a broad negative correlation between SCAF4‐POLR2A expression and activated T cell levels, as well as a positive correlation with resting T cell levels. This phenomenon was also observed in NK cell populations as elevated SCAF4‐POLR2A expression was negatively correlated with activated natural killer (NK) cells and positively correlated with inhibitory NK cell populations (Figure 7M). These findings further underscore the broad clinical potential of targeting the SCAF4‐POLR2A axis with PT_f_‐SRiApt, which could reprogram the tumor microenvironment and potentiate the efficacy of immune checkpoint inhibitors and other immunotherapeutic strategies.

## 3 Conclusion

TNBC is the most aggressive and diverse breast cancer subtype, accounting for 15%–20% of cases. Characterized by the absence of ER, PR, and HER2 receptors, TNBC is unresponsive to common HER2‐targeted and hormonal therapies.^[^ ^37^, ^38^, ^39^ ^]^ This makes the development of new therapeutic targets and effective treatments for TNBC an urgent priority. In this study, we utilized inhibitory aptamers to target the unique transcriptional landscape in TNBC and established them as versatile chemical tools for illustrating the potential of protein‐protein interaction targets for therapeutic intervention.

We presented PT_f_‐SRiApt as a first‐in‐class aptamer‐based chemical tool that selectively disrupts the “undruggable” protein‐protein interaction between SCAF4 and POLR2A, uncovering a previously unrecognized chemical intervention target in TNBC treatment. Extensive in vitro and in vivo analyses demonstrated that PT_f_‐SRiApt preferentially suppresses the proliferation of “transcriptionally addicted” TNBC cancer cells with heightened SCAF4 and POLR2A expression. Mechanistically, this suppression involved the promotion of RNA polymerase II transcription termination, activation of the MHC class II pathway, cell cycle arrest, and elevated immune infiltration, all of which synergistically contribute to a marked inhibition of breast cancer progression in murine models.

Moreover, leveraging PT_f_‐SRiApt, we further revealed the critical role of the SCAF4‐POLR2A interaction in intrinsic immune response and tumor microenvironment. Blocking this interaction with PT_f_‐SRiApt activates the MHC pathways, promotes leukocyte infiltration, and enhances their antitumor immunity. Additionally, co‐expression analysis of SCAF4 and POLR2A further supports the predictive role of their interaction in immunotherapy. These findings uncover a novel function of the SCAF4‐POLR2A interaction in TNBC and highlight the potential of PT_f_‐SRiApt in tumor immunotherapy.

Interestingly, while PT_f_‐SRiApt could bind to both SCAF4 and SCAF8 and inhibit their bindings to POLR2A due to the structural similarity of their CID domains, we observed that the expression level of SCAF8‐POLR2A did not correlate with SRiApt sensitivity. This supports the distinct functional roles of SCAF4 and SCAF8 in transcription regulation.^[^ ^7^ ^]^ Consequently, PT_f_‐SRiApt not only holds promise as a therapeutic agent but also serves as a valuable tool for distinguishing the functional specificities of SCAF4 and SCAF8, further highlighting the broader potential of aptamers in elucidating protein‐protein interactions and their biological roles.

## 4 Experimental Section

### Fluorescence Polarization

Experimental procedures were carried out using a Perkin Elmer EnVision 2104 Multilabel Reader (BioTek, USA, v1.14) equipped with 485 nm excitation and 535 nm emission filters for the FITC. FP measurements were performed using a Bioland 96‐well plate (product #PB06‐96S). Both parallel and perpendicular fluorescence intensity (Fǁ and F⊥) relative to linearly polarized excitation light were determined to calculate the FP signal. The affinity experiments were conducted in three biological replicates, each containing three technical replicates. The average affinity value was determined using the GraphPad Prism 7 program (GraphPad Software, Inc., USA) through curve fitting. The non‐linear fit model and the one‐site specific binding to fluorescent population ratio are used for these calculations at the respective aptamer concentrations. The FP assay buffer was constituted of 30 mM MES buffer pH 6.5, 25 mm NaCl, 2 mm β‐ME, 1 mm CHAPS, and 0.002 mg mL^−1^ BSA.

### Competition Assays of SRiApt

Competition assays of PT‐modified SRiApt for SCAF4‐POLR2A interaction were performed using Fluorescence Polarization (FP) assays. In brief, FP signals resulting from the interaction were detected by combining FITC‐labeled peptides with SCAF4 (40 nm) in the FP competition buffer (30 mm MES pH 6.0, 25 mm NaCl, 2 mm β‐ME, 1 mm CHAPS, and 0.002 mg mL^−1^ BSA). Each well of a 96‐well plate was loaded with 100 µL of the assay solution. SRiApt or PT‐modified SRiApt were serially diluted to varying concentrations and added to each well for the competition assay. Experiments were performed in three biological replicates, each containing three technical replicates, and the average inhibition constant values were determined by performing curve fitting and data analysis using GraphPad Prism 7 (GraphPad Software, Inc., USA) with the Log(inhibitor) versus normalized response model.

### Cell Lines and Mice

TNBC cell lines were obtained from ATCC and cultured according to ATCC's recommendations with Dulbecco's modified Eagle's medium (DMEM; Gibco) or RPMI 1640 (YESEN, 41402ES76) with 10% FBS (Sunrise, SR100180.03) and 1% penicillin/streptomycin (Biosharp, BL505A). Cells were cultured at 37 °C in an incubator containing 5% CO_2_ and were passaged no more than 25 times. Six‐ to eight‐week‐old female Balb/c mice (GemPharmatech Co., Ltd., Nanjing, China) or Balb/c nude mice (Qizhen, Hangzhou, China) were housed in a temperature‐controlled, pathogen‐free room. All animal procedures were performed in accordance with the approved protocols and guidelines of the Hangzhou Institute of Medicine Animal Experiment Ethics Committee (Hangzhou, China). The Animal Experiment Ethics number: AP2024‐08‐0169, AP2024‐10‐0309, AP2025‐04‐0495.

### Cell Cycle Assay

4T1 Cells were plated in 12‐well plates and treated with PT_f_‐SRiApt aptamer or CS aptamer (0.5 µm) as a control for 24 h. A Cell Cycle and Apoptosis Analysis Kit (Beyotime, C1052) was used for the following analysis. Cells were fixed in 1 mL 75% ethanol for 1 h at 4 °C and stained with Propidium (PI) solution for 30 min in a 5% CO_2_ incubator at 37 °C. Cells were analyzed using a Thermo Attune NxT flow cytometer. The number of live cells in each stage was determined by FlowJo software (version 10.8.1).

### Cell Colony Assay

4T1 cells or HCC1806 cells were plated in 12‐well plates and treated with PT_f_‐SRiApt aptamer or CS aptamer (5 µm) as a control for 5 days. After treatment, cells were washed with PBS and fixed in 4% multigrade formaldehyde (Biosharp, BL539A) for 10 min at room temperature. Cells were then washed with ddH_2_O twice and stained with Crystal Violet Staining Solution (Beyotime, C0121) for 10 min. After staining, cells were washed with ddH_2_O. Cells were observed under the scan and analyzed by ImageJ (version 1.8.0).

### Cell Proliferation Assay

Different cells were plated at different cell numbers per well in 96‐well plates with PT_f_‐SRiApt aptamer or CS aptamer for 3–5 days of treatment (4T1: 5000 cells per well; EMT6: 1500 cells per well; HCC1806: 10000 cells per well; MDA‐MB‐231: 8000 cells per well; MDA‐MB‐468: 15000 cells per well; CAL‐120: 10000 cells pre well; Hs‐578: 5000 cells per well; SUM159PT: 5000 cells per well). Imaged and analyzed with the Incucyte SX5 live cell imaging device (Sartorius, Germany).

### Tumor Treatment

1 × 10^6^ viable 4T1 breast cancer cells (in 50 µL of PBS) were subcutaneously injected into the backs of Balb/c mice. When tumors grew to measurable sizes, they were randomly grouped according to experimental needs. The mice were given PBS, PT_f_‐SRiApt aptamer, or CS aptamer (1 mg kg^−1^ in 50 µL of PBS) every day. The tumor volume was measured in a blinded manner using slide calipers and was calculated using the following formula: tumor volume (mm^3^) = length × (width)^2^ /2.

### Intratumor Infiltrating T‐Cell Analysis

Tumors were collected from the mice and minced into fine pieces in a digestion buffer containing 2% FBS and collagenase IV (2 mg mL^−1^, Gibco, 17104019). The samples were incubated in the digestion buffer at 37 °C for 1 h with a shaker, filtered through a 100‐µm filter, hemolyzed in hemolysis buffer (Solarbio, R1010), and washed twice with PBS. The collected cells were stained with the following fluorescent‐labeled antibodies: CD45 (Clone: 30‐F11, Cat: # 563891, BD Horizon), CD3e (Clone:145‐2C11, Cat: # 562600, BD Horizon). All flow cytometry was performed on Agilent NovoCyte Quanteon(Agilent Technologies, Inc., USA), and the analyses were performed using NovoExpress software(version 1.6.2).

### Anti‐PD‐1 Antibody and PT_f_‐SRiApt Aptamer Combination Treatment

7.5 × 10^5^ viable 4T1 breast cancer cells (in 50 µL of PBS) were subcutaneously injected into the backs of Balb/c mice. Tumor‐bearing mice were intratumorally treated with PBS or PT_f_‐SRiApt aptamer (0.5 mg kg^−1^ in 50 µL of PBS) every day, and intraperitoneally treated with InVivoMAb anti‐mouse PD‐1 (CD279) (200 µg in 50 µL of PBS, Cat: # BE0273, Clone: 29F.1A12, BioXcell) every 2 days. The tumor volume was measured every day.

### Pharmacokinetic (PK) / Pharmacodynamic (PD) Assay

1 × 10^6^ viable 4T1 breast cancer cells (in 50 µL of PBS) were orthotopically into the fourth mammary fat pad of BALB/c mice. When the tumor volume in mice reached ≈200 mm^3^, Cy7 solution or PT_f_‐SRiApt‐Cy7 (3 nmol per mouse) was administered via intratumoral injection. The mice were photographed at 0, 2, 4, 6, 8, 10, 12, 24, 48, 72, and 96 h via the In vivo imaging system IVIS Spectrum CT (Caliper Life Sciences, Inc.) utilized to monitor the time‐course biodistribution of Cy7 fluorescence in accordance with the manufacturer's protocols. Living image Software (4.8.2 version) was used to measure the average radiance of the region of interest (ROI). The half‐life (t1/2) of the Cy7 solution or PT_f_‐SRiApt‐Cy7 was determined by fitting the data to a plateau followed by a one‐phase decay model via GraphPad Prism 8 (GraphPad Software, Inc., USA).

### Complete Blood Count and Biochemical Analysis

Blood was sampled from the hearts of treated mice on day 11. Whole blood samples pre‐treated with heparin were measured by TEK8500 automatic hematology animal blood analyzer (TECOM, Jiangxi, China), while serum levels ALT, AST, UA, CR, TBII, ALB, and BUN were immediately measured using an automatic biochemical analyzer 7180 (HITACHI, Japan)

### Differentially Expressed Genes Identification and Gene Set Enrichment Analysis

Differentially expressed genes for HCC1806 bulk mRNA sequencing were determined using R package DESeq2 (v1.40.2). Differentially expressed genes for single‐cell data were determined using the FindMarkers function in package Seurat (v4.4.0) with thresholds set to negative infinity. Significantly differentially expressed genes were selected by setting the threshold to *p* < 0.05 and the absolute value of Log_2_(FC) > 1.

Functional analysis was performed using R package fgsea (v1.26.0). Curated Molecular Signatures Database (MSigDB) Gene Ontology (GO), Reactome, and Chemical and Genetic Perturbations of human gene sets were assessed for HCC1806 bulk sequencing data. GO mouse gene sets were assessed for 4T1 mouse model single‐cell sequencing. Default unadjusted *p* values were used and displayed in the figures.

### Gene Scoring and Immune Deconvolution

SCAF4/POLR2A expression score was calculated by taking the mean of scaled expression of both gene expressions. Each gene expression was scaled to a distribution with a mean of zero and a standard deviation of one. Immune deconvolution was performed with the Cibersort option with default parameters using the R package IOBR (v0.99.8). Pearson correlation was calculated using cor.test function from the R stats package (v4.3.3), *p* values were calculated using asymptotic confidence intervals based on Fisher's z transformation.

### Single‐Cell Sequencing Data Processing and Analysis

Seurat standard workflow was implemented using the R package Seurat (v4.4.0). Cells were filtered using the following parameters: nFeatures_RNA > 200, nCount_RNA < 30 000, percent.mt < 10. Single‐cell data was clustered using 0.5 resolution, six‐cell subtypes were identified in total using markers: epithelial cell (Epcam, Krt18, Krt8), endothelial cell (Pecam1, Egfl7, Flt1), macrophage (Itgam, Csflr, Cd68, C1qa), T cell (Cd3e, Cd3g), neutrophil (Csf3r, Cd33, S100a8), and cancer‐associated fibroblasts (Col1a1, Col12a1, Pdpn).

### Statistical Analysis

Single‐cell RNA sequencing data was normalized and scaled using standard Seurat v4.4.0 workflow. All results are expressed as the mean ± standard deviation (SD). ANOVA with Tukey's multiple comparisons test was performed to calculate *p*‐values when comparing continuous tumor growth. Two‐tailed Student's t‐test was performed to calculate *p*‐values when comparing groups of biological replicates. Two‐tailed Wilcoxon test from R package ggpubr (0.6.0) was performed to calculate *p*‐values when comparing gene expression across groups. The standard permutation test from R package fgsea (v1.26.0) was used to calculate *p*‐values in GSEA. All statistical analyses were performed in R and GraphPad Prism. In all cases, significance was defined by *p* < 0.05.

## Conflict of Interest

The authors declare no conflict of interest.

## Supporting information



Supporting Information

## Data Availability

The data that support the findings of this study are available in the supplementary material of this article.
