# Supplementary material for: PTf‐SRiApt Targeting SCAF4‐POLR2A Interaction Suppresses Tumor Growth and Promotes Antitumor Immunity in Triple‐Negative Breast Cancer
Source: Adv Sci (Weinh). 2025 Jun 27;12(35):e00433. doi: 10.1002/advs.202500433 (PMC12463048; doi:10.1002/advs.202500433)
Supplement: Supplementary file 1 — Supporting Information [file ADVS-12-e00433-s001.docx]

Supporting Information

PT_f_-SRiApt Targeting SCAF4-POLR2A Interaction Suppresses Tumor Growth and Promotes Antitumor Immunity in Triple-Negative Breast Cancer

Liyan Fei, Yichun Pan, Jie Zhai, Tongqing Li, Yuxin Zhou, Sheyu Zhang, Juan Wei, Qian Hu, Xueying Liu, Lu Guo, Weizhu Wu, Yong Wei,* Qin Wu,* Weihong Tan*

**Supplementary Figures**

**
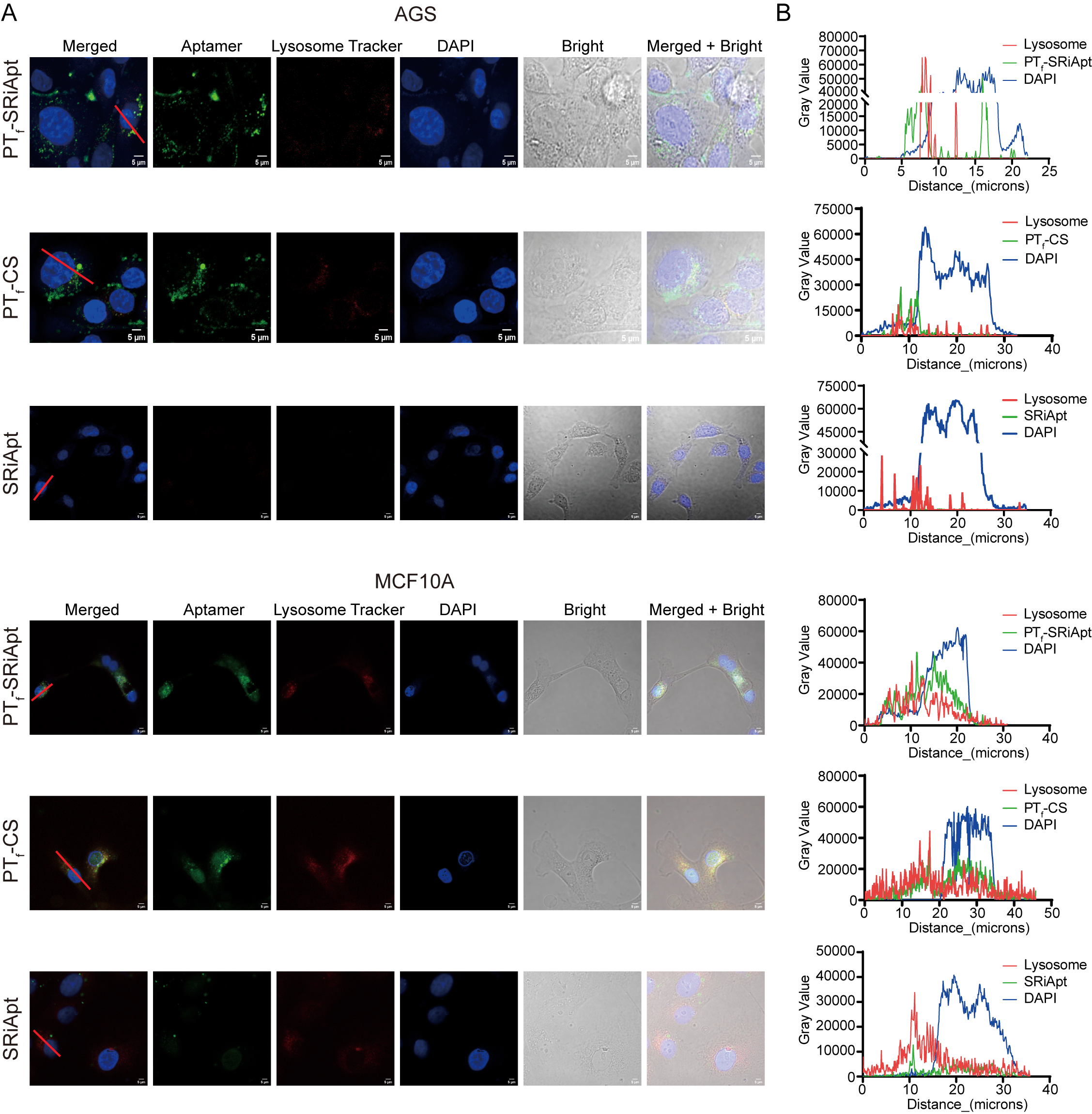
**

Figure S1. Cellular intake of PT_f_-SRiApt in cell lines using confocal microscopy. A) Confocal microscopy images depicting the endocytosis of PT_f_-SRiApt, PT_f_-CS, and SRiApt in AGS and MCF10A cells. Cells were treated with 1 µm of corresponding FAM-labelled oligonucleotide for 24 hours at 37℃, followed by co-staining with a lysosome tracker and DAPI after formaldehyde fixation. Scale bar = 5 µm. B) Quantification of fluorescence intensities from FAM-PT_f_-SRiApt (top), FAM-PT_f_-CS (middle), and FAM-SRiApt (bottom), lysosome tracker, and DAPI in gray scale values following the red line in panel A in AGS and MCF10A cells, demonstrating intracellular distribution and lack of lysosomal accumulation.

**
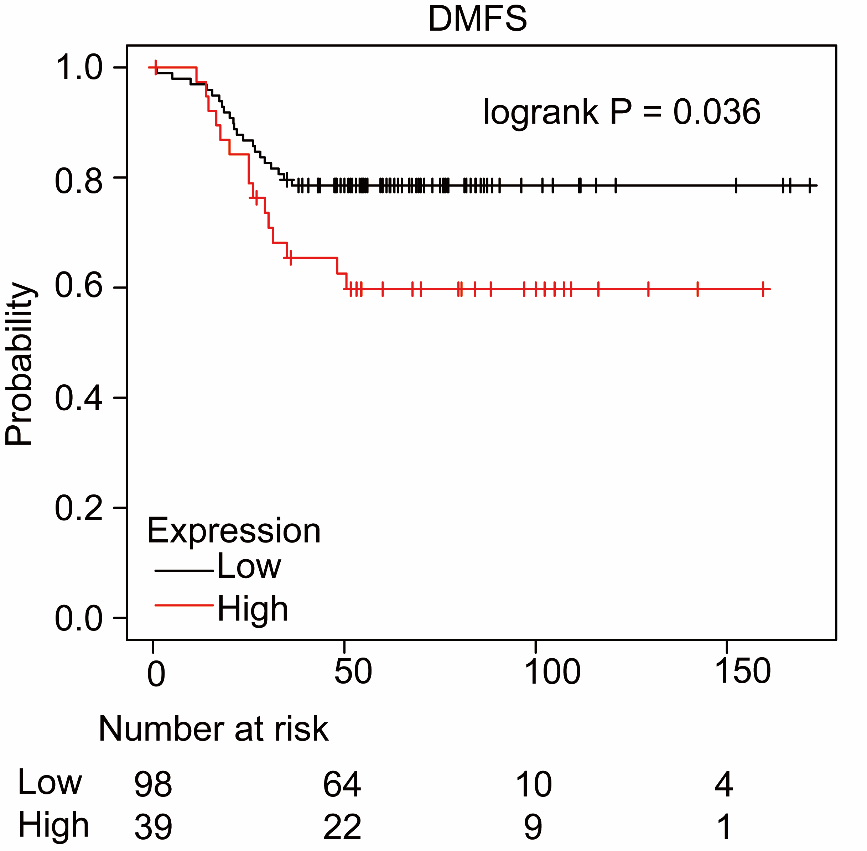
**

Figure S2. Kaplan-Meier survival analysis showing reduced Distant Metastasis-Free Survival (DMFS) in breast cancer patients with high co-expression of SCAF4 and POLR2A.

**
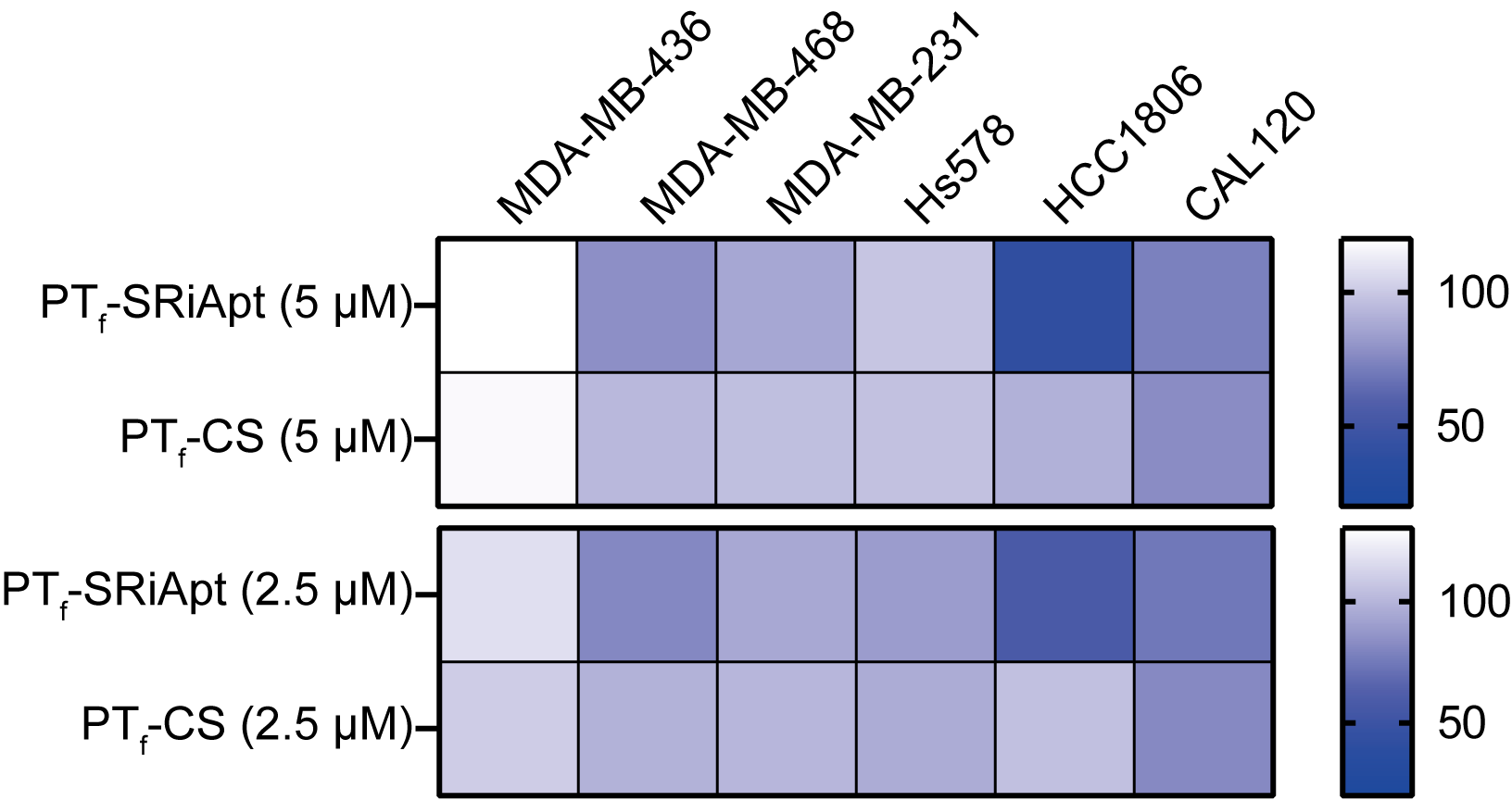
**

Figure S3. Growth curves of six breast cancer cell lines treated with PT_f_-SRiApt or PT_f_-CS in 5 µm or 2.5 µm for five days (n = 3). Data are presented as mean ± SD.


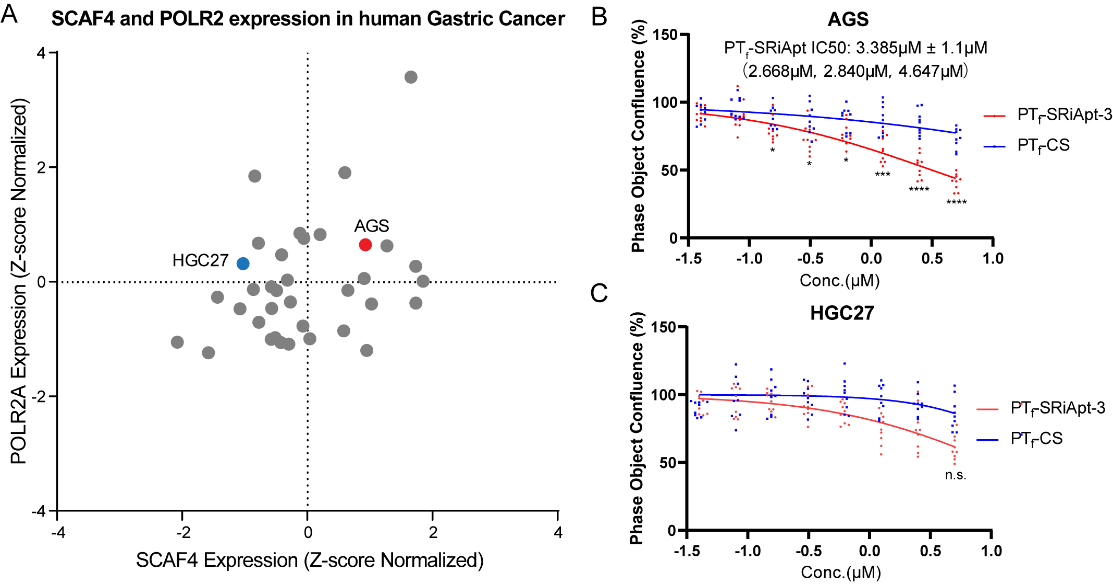


Figure S4. PT_f_-SRiApt treatment of gastric cancer cell lines. A) SCAF4 and POLR2A expression in gastric cancer cell lines. B) Growth curves of AGS cells treated with PT_f_-SRiApt or PT_f_-CS for five days (n = 3).C) Growth curves of HGC27 cells treated with PT_f_-SRiApt or PT_f_-CS for five days (n = 3). Data are presented as mean ± SD; **** indicates *p* < 0.0001, determined using two-tailed Student's t-test.


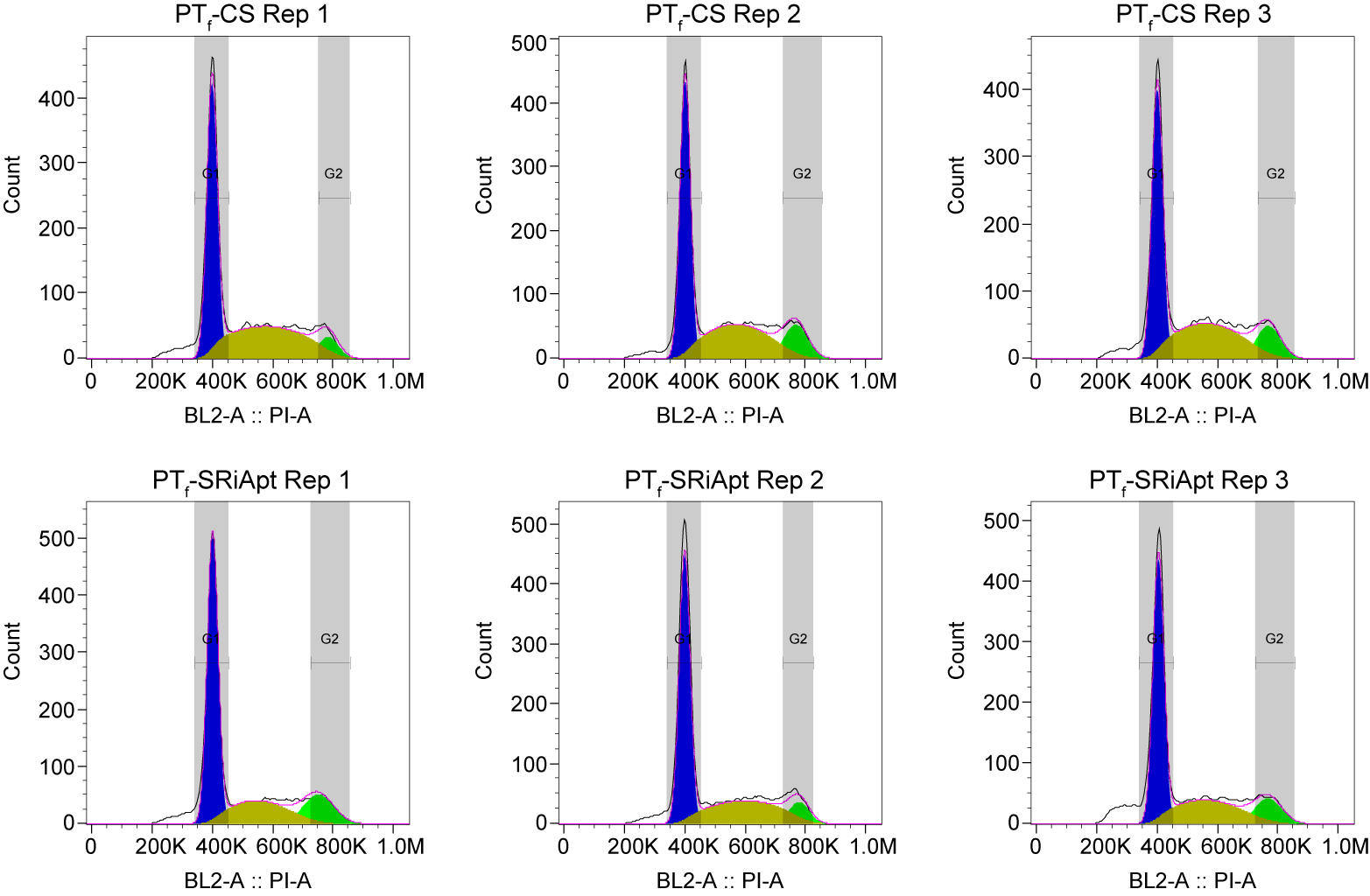


Figure S5. Cell cycle effects on HCC1806 cells treated with PT_f_-SRiApt or PT_f_-CS for five days (n = 3), using a Novocyte flow cytometry system and NovoExpress software (ACEA Bioscience Inc.).

**
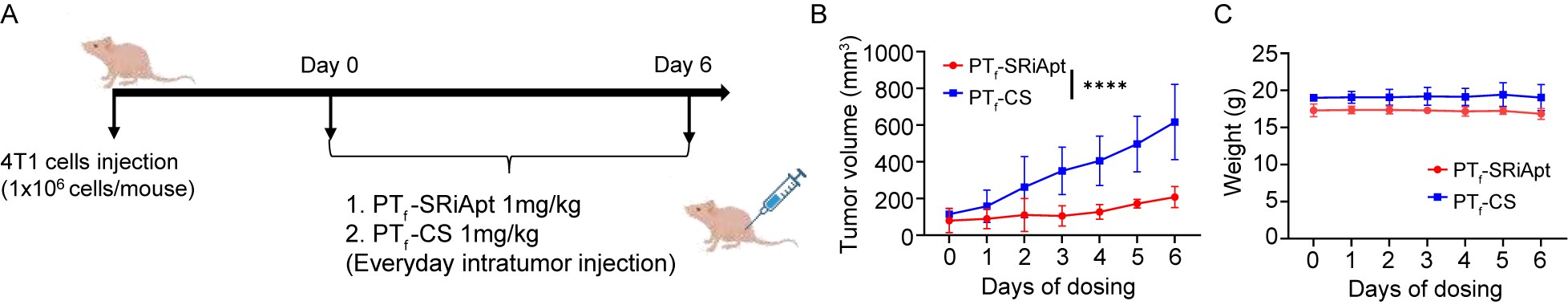
**

Figure S6. Anti-tumor efficacy of PT_f_-SRiApt in Breast cancer model. A) Schematic illustration of the treatment schedule with PT_f_-SRiApt or PT_f_-CS for immunodeficient nude mice. B) Averaged tumor volume curves of mice treated with PT_f_-SRiApt, or PT_f_-CS. Data represent mean±SD of tumor volumes calculated from the diameter of the tumor mass. Differences at day 6 were considered statistically significant if the *P* value was less than 0.05, as determined by the one-way ANOVA with Tukey’s multiple comparisons test. **** indicate *p* < 0.0001. C) Body weights of immunodeficient nude mice in the PT_f_-SRiApt, or PT_f_-CS groups. The data is presented as the mean ± SD.

**
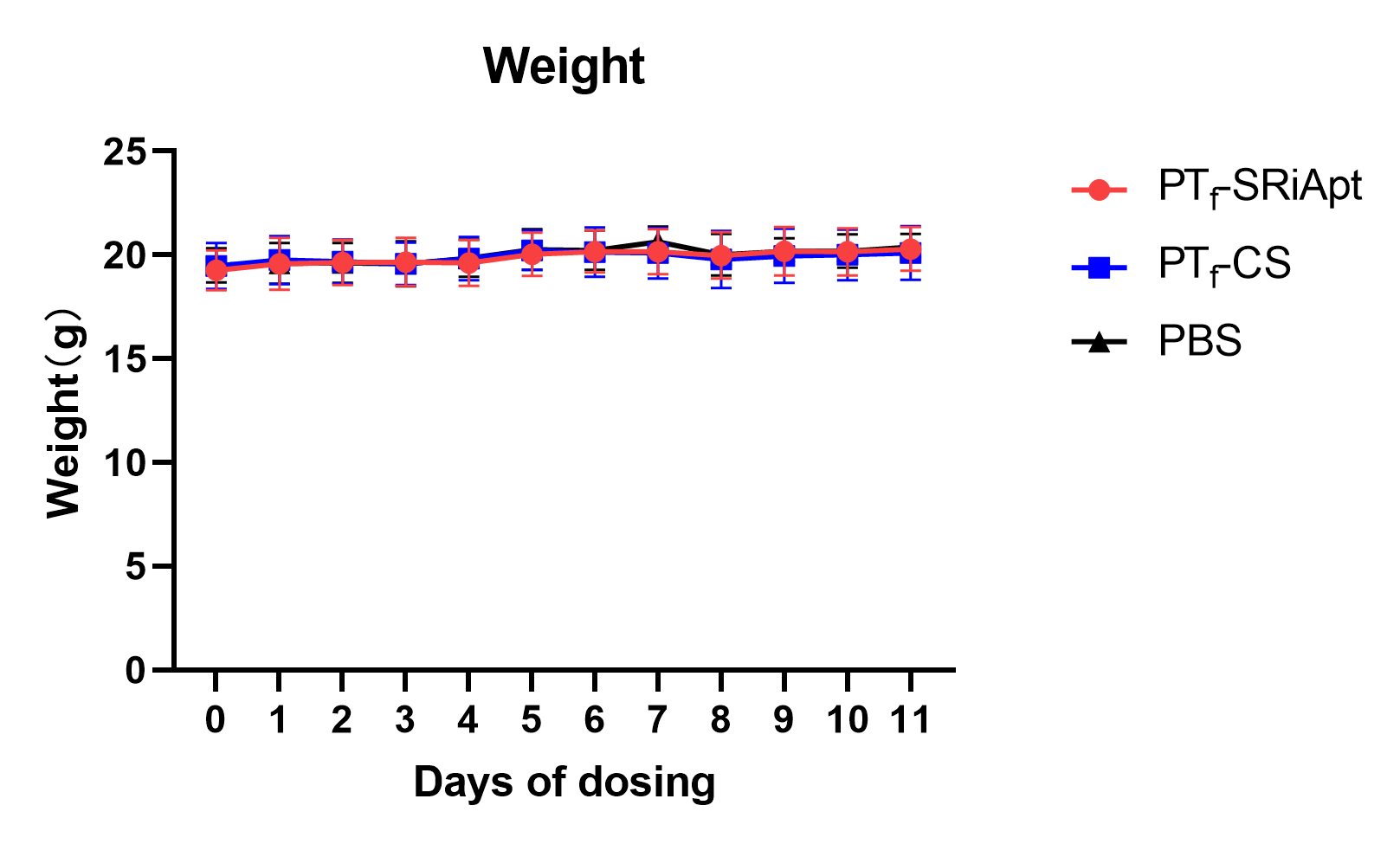
**

Figure S7. Body weights of BALB/c mice in the PBS, PT_f_-SRiApt, or PT_f_-CS groups. The data is presented as the mean ± SD.

**
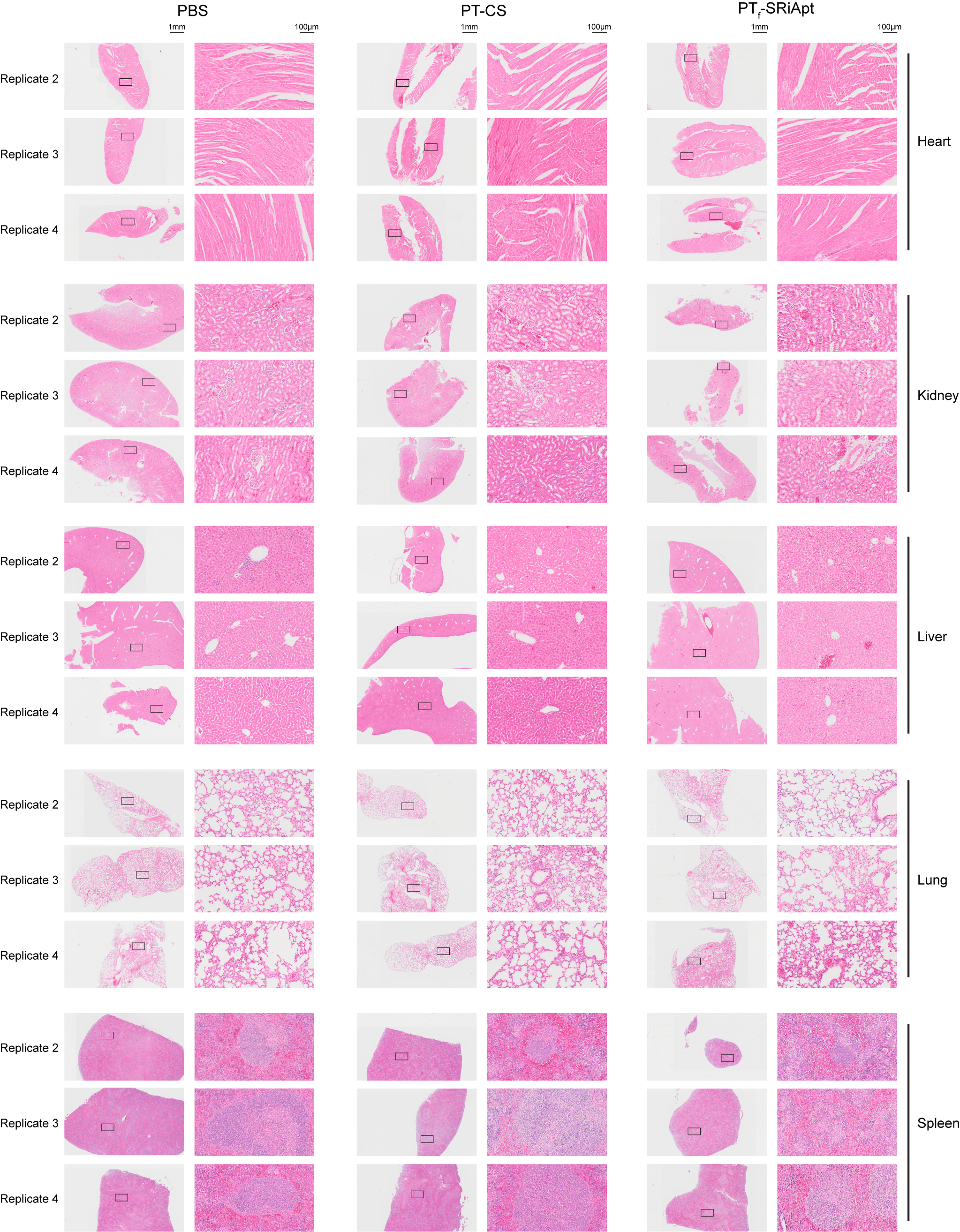
**

Figure S8. H&E stains of the main organs (heart, liver, spleen, lung, and kidney) treated with PT_f_-SRiApt, PT_f_-CS, or PBS control in BLAB/c mice replicates.

**
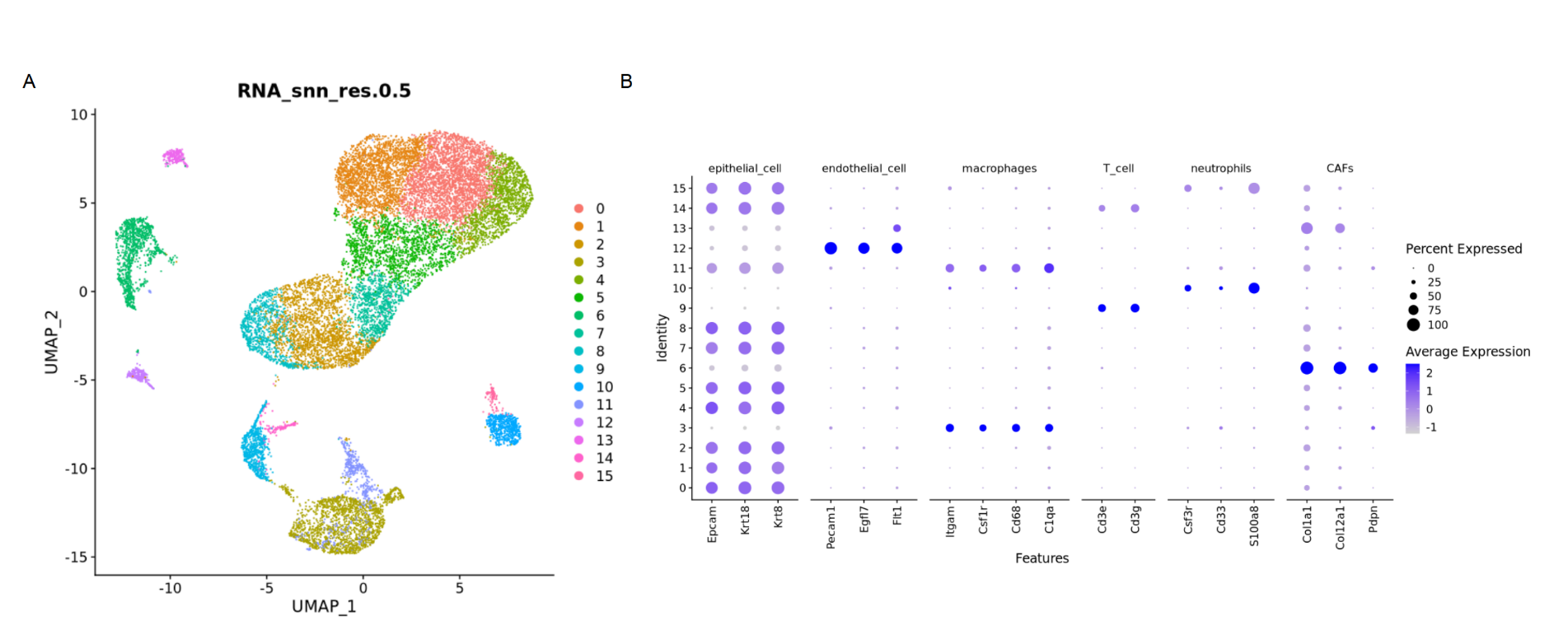
**

Figure S9. Clustering of single cells. A) UMAP plot showing cell populations identified within tumors from both the PT_f_-SRiApt and control groups. Clustering of single cell data with resolution of 0.5 results in 16 clusters. B) Cellular markers used for cell subtype clustering.

**
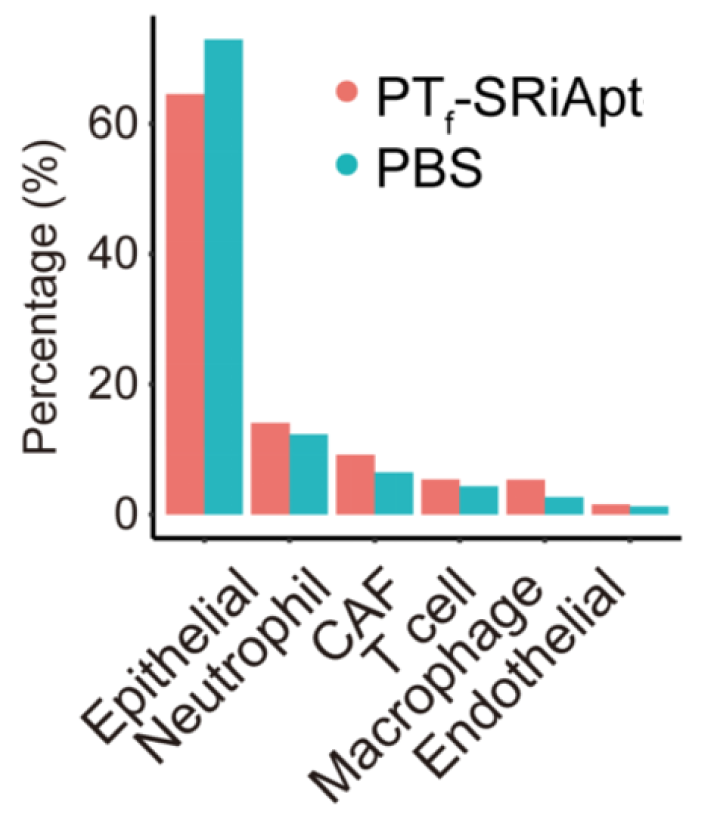
**

Figure S10. Percentages of the different cell populations in PT_f_-SRiApt or PBS treated groups.

**
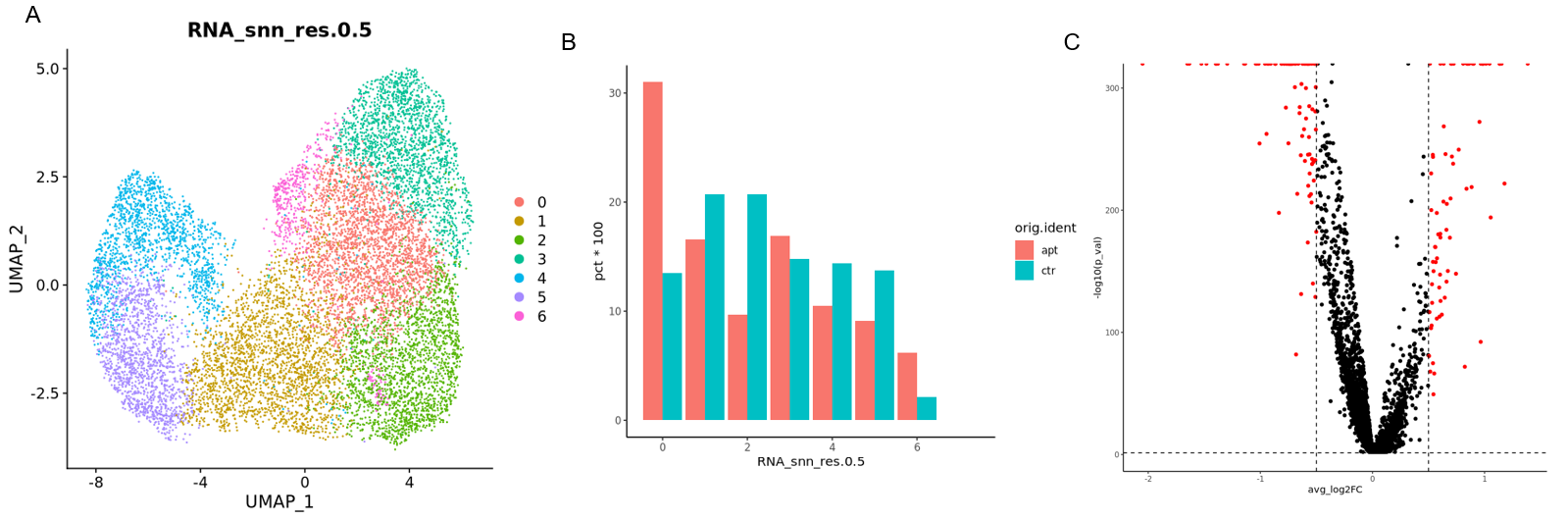
**

Figure S11. Subclustering of epithelial cells. A) Clustering of epithelial cell data with resolution of 0.5 results into seven clusters. B) Variations of epithelial cell clusters after PT_f_-SRiApt treatment. C) Differentially expressed genes comparing treatment sensitive epithelial cells to treatment resistant epithelial cells.

**
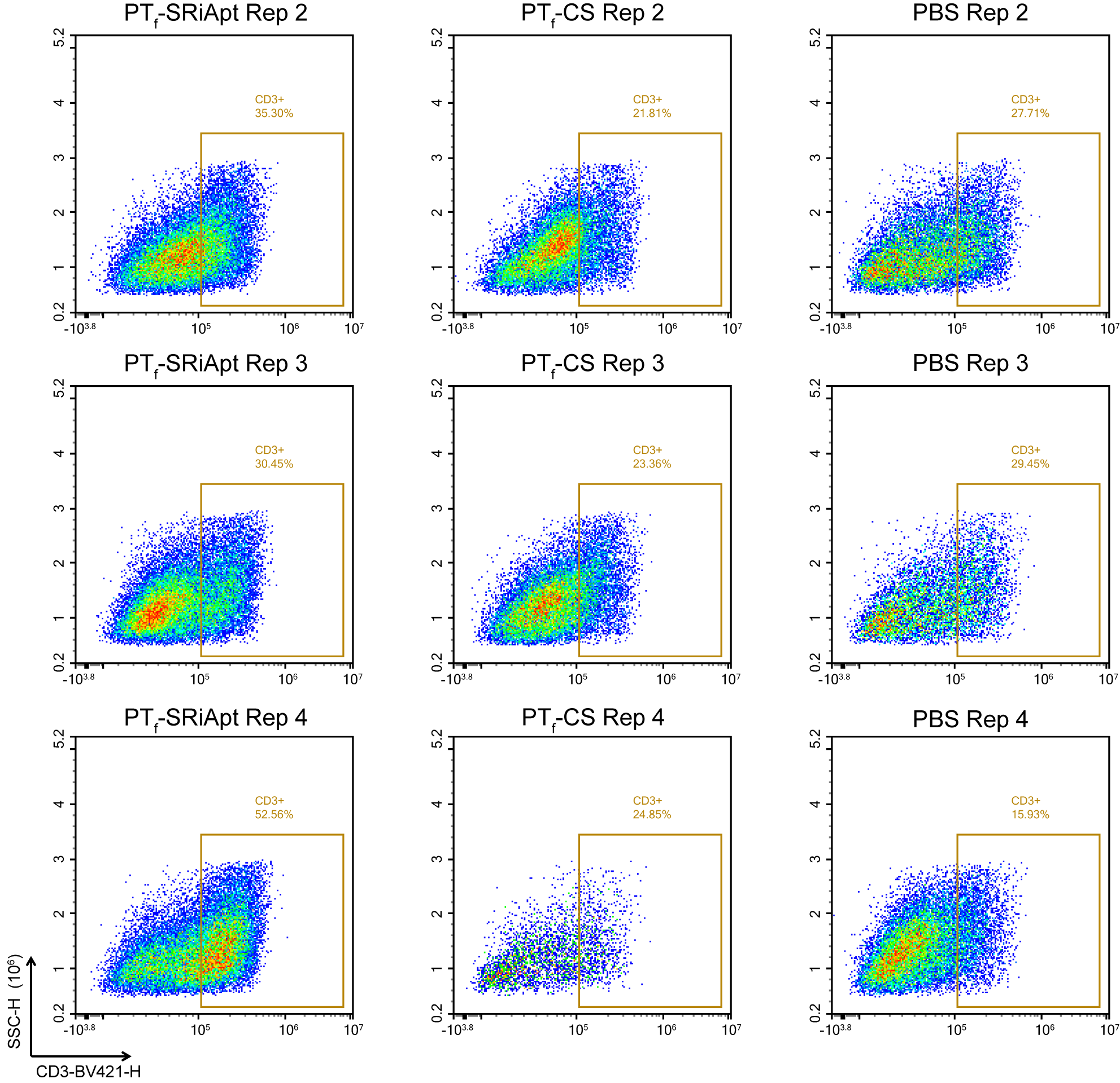
**

Figure S12. Representative flow cytometric analysis of T cells in tumors treated by PT_f_-SRiApt, PT_f_-CS or PBS, replicates 2 to 4.

**
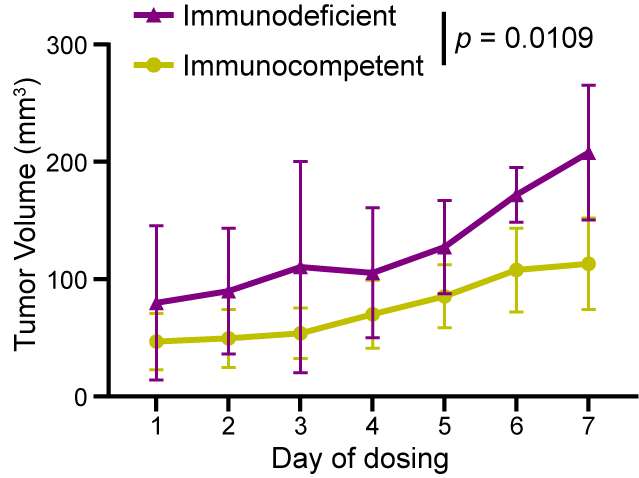
**

Figure S13. Comparison of average tumor volume curves between immunodeficient nude mice and immunocompetent BALB/c mice treated with PT_f_-SRiApt. Data are derived from Fig. 4B and SI Fig. 4B and represent mean ± SD of tumor volumes. Statistically significant differences on day 6 were calculated using one-way ANOVA with Tukey’s multiple comparisons test (p = 0.0109).

**
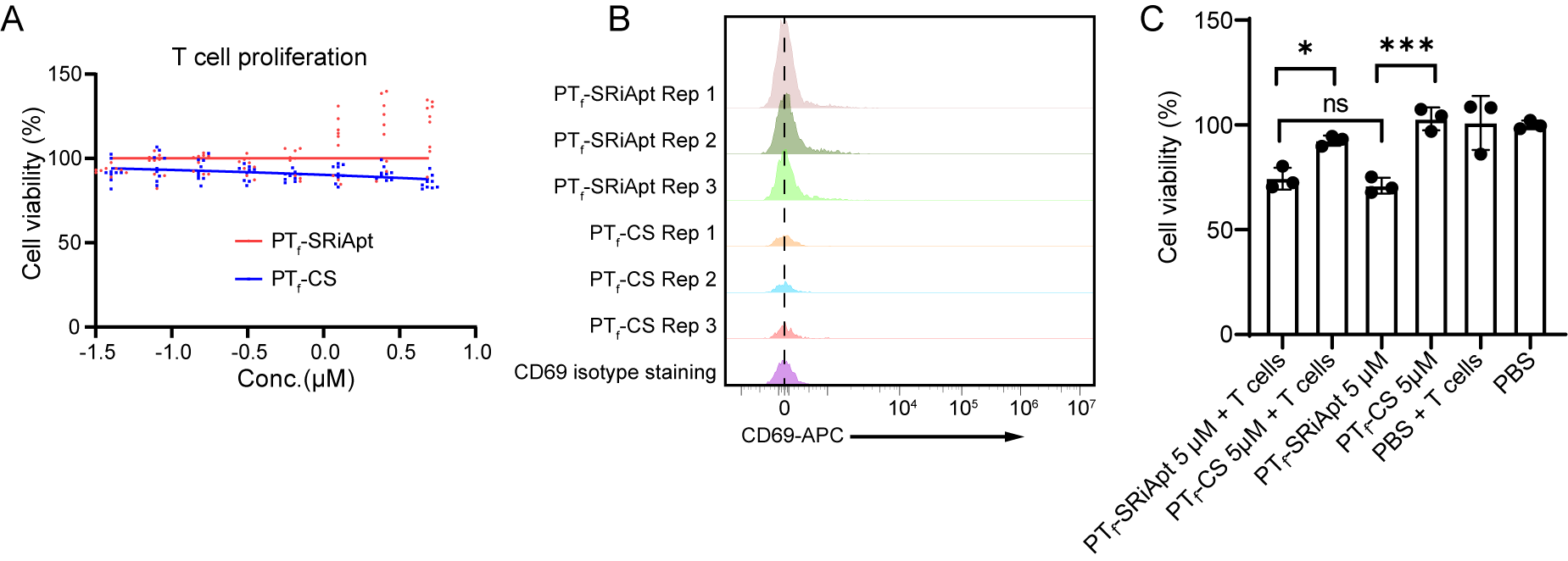
**

Figure S14. T cell proliferation, activation and tumor-killing after PT_f_-SRiApt treatment. A) *In vitro* T cell proliferation after PT_f_-SRiApt treatment. B) *In vitro* T cell activation after PT_f_-SRiApt treatment. C) *In vitro* tumor-killing efficacy of T cells after PT_f_-SRiApt treatment.


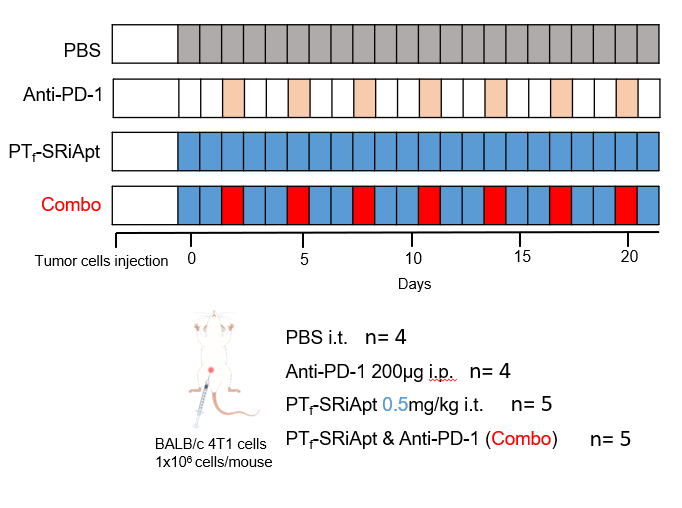


Figure S15. Schematic illustration of the treatment schedule with PBS, anti-PD-1, PT_f_-SRiApt, and combination therapy.

**Methods and Materials**

**Protein expression and purification**

The Human SCAF4-CID domain fragment comprising residues 1-139 was purified according to the previous report (Blocker-SELEX). Shortly, the recombinant protein was expressed in BL21 (DE3) cells and induced with 0.5 mm isopropyl-β-D-thiogalactopyranoside (IPTG) at 16°C overnight. Cells were collected via centrifugation, resuspended in lysis buffer containing DPBS buffer, 0.05 mm EDTA, and 5 mM imidazole, and sonicated. The supernatant was obtained after centrifugation at 16000 x g for 1 hour and further purified via the Ni-NTA Beads Gravity Column (Changzhou Smart-Lifesciences Biotechnology Co., China). His-tag was cleaved using TEV Protease, and the protein was subsequently purified using a Superdex75 gel-filtration column (GE Healthcare, Chicago, IL, USA).

**Confocal imaging**

4T1 cells, AGS cells and MCF10A cells were seeded and treated with PT_f_-SRiApt aptamer, PT_f_-CS aptamer or SRiApt aptamer (1 μm) for 24 hours. After treatment, cells were washed with PBS and then incubated with lysotracker (50nM, YESEN, 40739ES50) for 1 hour in a 5% CO2 incubator at 37 °C. After incubation, cells were washed with PBS twice and fixed in 4% multigrade formaldehyde (Biosharp, BL539A) for 15 minutes at room temperature. Cells were then washed with PBS twice and blocked in 5% bovine serum albumin (Biosharp, BS114) for 45 minutes at room temperature. Cells were washed with PBS twice, and Antifade Mounting Medium with DAPI (Beyotime, P0131) was added and incubated for 1 minute. Images were detected on a single-photon confocal microscope (NIKON, A1 HD25) and analyzed by ImageJ (version 1.8.0).

**HE staining**

The hearts, livers, spleens, lungs, and kidneys from mice were harvested and preserved in 4% paraformaldehyde (Biosharp, BL539A) at 4°C for 24 hours. Following fixation, the samples were processed for dehydration and paraffin embedding by the Tissue-Tek VIP® 6 AI (SAKURA, Osaka, Japan) and the HistoCore Arcadia modular tissue embedding system (Leica, Germany). Representative sections were obtained by trimming the paraffin blocks until the tissue surface was adequately exposed, using the HistoCore AUTOCUT (Leica, Germany). The tissues were then sectioned at a thickness of 5 mm and stained with haematoxylin and eosin (H&E) through the DRS-Prisma-P-JCS&Film-JC2 system (SAKURA, Osaka, Japan). The images of the stained sections were captured by the Olympus VS200 tissue observation system (Olympus, Tokyo, Japan).

**T Cell isolation**

The T cells were isolated from human peripheral blood mononuclear cells (STEMCELL, Vancouver, Canada) as the protocols of EasySep™ Human T Cell Isolation Kit (STEMCELL, 17951, Vancouver, Canada) and cultured with cultured RPMI 1640 (YESEN, 41402ES76) contain 10% FBS (Sunrise, SR100180.03) and 1% penicillin/streptomycin (Biosharp, BL505A) and 0.05% IL-2 protein (Gibco^TM^, AF-200-02-10UG, USA). Cells were cultured at 37°C in an incubator containing 5% CO_2_.

**T cell proliferation and cytotoxicity assay**

T cells were seeded in 96-well plates at 50,000 cells per well and incubated for 3 days with PT_f_-SRiApt or CS aptamers at a range of concentrations obtained through serial dilution. For the T cell cytotoxicity assay, HCC1806 cells were seeded in 24-well plates at a density of 50,000 cells per well. 24 hours later, an equal number of T cells were co-cultured with HCC1806 cells and treated with PT_f_-SRiApt or CS aptamer for 24 hours. The sensitive colorimetric assays for the determination of the percentage of viable cells in cell proliferation assays, using Cell Counting Kit-8 (CCK-8) (Biosharp, BS350B). The data were determined by performing curve fitting and analysis using GraphPad Prism 8 (GraphPad Software, Inc., USA) with the Log(inhibitor) vs. normalized response model.

**T cells activation assay**

T cells were seed at 1 × 10^6^ per well in 6-well plates and treated with PT_f_-SRiApt aptamer or CS aptamer (5 μm) for 24 hours. Later, cells were collected and stained with APC anti-human CD69 (clone: FN50, Cat: # 985206, Biolegend, USA) or APC Mouse IgG1, κ Isotype Ctrl (clone: MOPC-21, Cat: # 981806, Biolegend, USA). All flow cytometry was performed on a CytoFLEX Flow Cytometers (Beckman Coulter, Inc., USA) and the analyses were performed using FlowJo software (version 10.8.1).

**Bulk mRNA library construction and sequencing**

Sequencing library prepared using 1 μg total RNA. The poly(A) mRNA isolation was performed using Oligo(dT) beads. The mRNA fragmentation was performed using divalent cations and high temperature. Priming was performed using Random Primers. First strand cDNA and the second-strand cDNA were synthesized. The purified double-stranded cDNA was then treated to repair both ends and add a dA-tailing in one reaction, followed by a T-A ligation to add adaptors to both ends. Size selection of Adaptor-ligated DNA was then performed using DNA Clean Beads. Each sample was then amplified by PCR using P5 and P7 primers and the PCR products were validated. Then libraries with different indexs were multiplexed and loaded on an Illumina HiSeq/ Illumina Novaseq/ MGI2000 instrument for sequencing using a 2x150 paired-end (PE) configuration according to manufacturer’s instructions. Sequenced fastq data were processed by Cutadapt (v1.9.1, phred cutoff: 20, error rate: 0.1, adapter overlap: 1bp, min. length: 75, proportion of N: 0.1) to ensure quality. Clean data were then aligned to human reference genome via software Hisat2 (v2.2.1).

**Single-cell mRNA library construction and sequencing**

Samples were dissociated into single cells in dissociation solution (0.35% collagenase IV5, 2 mg/mL papain, 120 Units/ml DNase I) in 37 ℃ water bath with shaking for 20 min at 100 rpm. Digestion was terminated with 1× PBS containing 10% fetal bovine serum (FBS, V/V), then pipetting 5-10 times with a Pasteur pipette。The resulting cell suspension was filtered by passing through 70-30um stacked cell strainer and centrifuged at 300g for 5 min at 4°C. The cell pellet was resuspended in 100ul 1× PBS (0.04% BSA) and added with 1 ml 1× red blood cell lysis buffer (MACS 130-094-183, 10×) and incubated at room temperature or on ice for 2-10 min to lyse remaining red blood cells. After incubation, the suspension was centrifuged at 300g for 5 min at room temperature. The suspension was resuspended in 100 μl Dead Cell Removal MicroBeads (MACS 130-090-101) and remove dead cells using Miltenyi® Dead Cell Removal Kit (MACS 130-090-101). Then the suspension was resuspended in 1× PBS (0.04% BSA) and centrifuged at 300 g for 3 min at 4 °C (repeat twice). The cell pellet was resuspended in 50 μl of 1× PBS (0.04% BSA). The overall cell viability was confirmed by trypan blue exclusion, which needed to be above 85%, single cell suspensions were counted using a haemocytometer/ Countess II Automated Cell Counter and concentration adjusted to 700-1200 cells/μl.

Single-cell suspensions were loaded to 10x Chromium to capture 5000 single cells according to the manufacturer’s instructions of 10X Genomics Chromium Single-Cell 3’ kit (V3). The following cDNA amplification and library construction steps were performed according to the standard protocol. Libraries were sequenced on an Illumina NovaSeq 6000 sequencing system (paired-end multiplexing run,150bp) by LC-Bio Technology co. ltd., (Hangzhou, China) at a minimum depth of 20,000 reads per cell. Quality control and upstream pre-processing was performed using 10X Cell Ranger software (v7.2.0) using mm10 mouse genome as alignment reference.
